# Business entrepreneurship in nursing care facilities in Brazil: an ecological study

**DOI:** 10.1590/1518-8345.7657.4584

**Published:** 2025-07-11

**Authors:** Matheus Moraes Silva, Jouhanna do Carmo Menegaz, Glenda Roberta Oliveira Naiff Ferreira, Josele de Jesus Quaresma Trindade Costa, Maria Eduarda Libório Martins, Allison Squires

**Affiliations:** 1 Universidade Federal do Pará, Faculdade de Enfermagem, Belém, PA, Brazil.; 2 Scholarship holder at the Coordenação de Aperfeiçoamento de Pessoal de Nível Superior (CAPES), Brazil.; 3 Universidade do Estado de Santa Catarina, Departamento de Enfermagem, Chapecó, SC, Brazil.; 4 Scholarship holder at the Conselho Nacional de Desenvolvimento Científico e Tecnológico (CNPq), Brazil.; 5 New York University, Rory Meyers College of Nursing, New York, NY, United States of America.

**Keywords:** Entrepreneurship, Nursing Services, Job Market, Private Duty Nursing, Professional Autonomy, Universal Health Coverage

## Abstract

to characterize and describe the spatial and temporal distribution of nursing care establishments in Brazil according to the National Registry of Health Establishments.

ecological study, using secondary data from the National Register of Health Establishments, aggregated by year, state and region. The variables extracted were: year of registration, gender of the nurse manager or administrator, number of nurses per establishment, type of establishment, main activity, level of care, legal nature and agreement. Descriptive statistics and temporal analysis were carried out using the Jointpoint program.

between 2003 and 2023, 340 nursing establishments were found with active registrations. The South and Southeast concentrated most of the establishments. There was a predominance of practices, specialized clinics and home care services. The temporal analysis indicated that between 2003 and 2021, there was an annual growth of 30.07% (p= 0.00). Considering the total period (2003-2023), there was an annual growth of 25.01%, indicating an upward trend in businesses over the years.

formalization and registration reflect the professionalization of nursing management. There has been an increase in registrations following the publication of resolutions by the Federal Nursing Council that support autonomous and/or liberal practice.

## Highlights

**(1)** The South and Southeast regions concentrate more nursing establishments. **(2)** Between 2003 and 2023 there was an annual growth of 25.01% in businesses. **(3)** Isolated practices and specialized clinics predominate. **(4)** The main activities are outpatient consultations, home care and immunization. **(5)** The majority accept private payment and private health plans.

## Introduction

Entrepreneurship in nursing is defined as the ability to identify opportunities, develop innovative projects and implement solutions for the continuous improvement of health care. The concept goes beyond the creation of companies, encompassing innovation, research and scientific advancement, enabling nurses to transform healthcare practices and meet the demands of society^(1)^.

On the international stage, entrepreneurship in nursing has been widely recognized for its potential to tackle complex challenges and promote innovation in the provision of health services^(2)^. A systematic review highlighted that nurse entrepreneurs identify gaps in healthcare systems and develop innovative services, such as specialized clinics, home care companies and independent practices, diversifying services and expanding access to care^(3)^.

In 2023, the American Nurses Association Enterprise Innovation highlighted that more than 300 nurse entrepreneurs led initiatives as agents of change. These actions included the development of medical devices, new models in the provision of care, business creation, research and educational specialties, highlighting the role of entrepreneurship in the advancement of the profession^(4)^.

In Brazil, the health sector is poised for expansion driven by demographic and political changes and transformations in consumer attitudes. The aging of the population, the increase in chronic diseases and the growth of the middle class have generated a growing demand for advanced health services and technologies^(5-7)^. In addition, Brazilians’ growing interest in preventive health is driving the development of sub-sectors such as health and wellness clinics^(8)^.

This context highlights entrepreneurial opportunities in the nursing field. The right to undertake and have one’s own business in nursing is supported by the Professional Practice Law No. 7,498/1986, which ensures nurses’ autonomy. In addition, business entrepreneurship in nursing stands out in the Brazilian scenario, especially with Resolution No. 568/2018 of the Federal Nursing Council (COFEN), which regulates the work of nurses in nursing clinics and offices^(9)^.

Thus, for the purposes of this resolution, a nursing clinic is defined as an establishment made up of offices and environments intended for individual, collective and/or home nursing care. On the other hand, the nursing office is defined as the exclusive physical area for consultations and other private activities of nurses^(9)^. These definitions reinforce the right to undertake and exercise the nursing profession autonomously and independently.

Nursing business entrepreneurs who own a care establishment, understood in this study as clinics or offices, need to meet a series of legal, tax, health and regulatory requirements before the Regional Nursing Council in order to act ethically and legally^(10)^. Healthcare establishments are delimited and permanent physical spaces in which human health actions and services are carried out under technical responsibility^(11)^.

In Brazil, the management of healthcare establishments is regulated by the National Register of Healthcare Establishments (CNES), a computerized system maintained by the Ministry of Health that centralizes information on all healthcare services in the country, whether public or private, with or without an agreement with the Unified Health System (SUS), and it is mandatory for all establishments offering human healthcare to have a register^(11)^.

Considering the lack of knowledge about business entrepreneurship in nursing care establishments, the scarcity of studies mapping its characteristics and temporal trends in Brazil, and the absence of unified data made available by the Regional Nursing Councils (CORENs) and COFEN on the panorama of these enterprises, this study aimed to characterize and describe the spatial and temporal distribution of nursing care establishments in Brazil according to the CNES.

## Method

### Study design

This is an ecological study, guided by the Strengthening the Reporting of Observational Studies in Epidemiology (STROBE) tool. The analysis and results of ecological studies are only applicable at the population level^(12)^. In this study, the units of analysis were the 27 states and 5 (five) administrative regions of Brazil.

### Setting

The study was conducted in Brazil, which has a territory of 8,510,417.77 km², divided into 26 states, a Federal District and 5,570 municipalities. The country is organized into five macro-regions: North, Northeast, Midwest, Southeast and South. By August 2022, the Brazilian population was estimated at 203,080,756 individuals^(13)^.

According to COFEN data, the country will have around 756,054 registered nurses by November 2024^(14)^. As for the proportion of nurses per 1,000 inhabitants in Brazil’s macro-regions, a study carried out in 2021, based on data from the nursing profile published by COFEN in 2016, showed the following statistics: North Region (1.6 per 1,000 inhabitants), Northeast Region (1.76 per 1,000 inhabitants), Midwest Region (2.25 per 1,000 inhabitants), Southeast Region (2.38 per 1,000 inhabitants) and South Region (1.77 per 1,000 inhabitants)^(15)^.

### Population and selection criteria

Data from nursing care establishments classified as Business Entities and Individuals (group), in which the professional nurse acted as manager or administrator, were included, considering only those with active registration with the CNES. Data with incomplete information or which did not meet the pre-established requirements was excluded.

It should also be noted that no time frame was defined for the selection of data, in order to include the largest number of establishments, so as to adequately measure the longitudinal evolution of entrepreneurship in nursing care establishments over time.

### Data collection

Data was collected using the CNES platform, in the Establishment Consultation section, using the keywords: “Nursing Clinic”, “Nursing Office”, “Nurse”, “Nurse”, “Nursing” and “Nurse”. This search methodology is justified by the absence of an option to search for establishments by health professional category. We therefore opted for this strategy in order to filter establishments related to nursing by their trade name or company name.

As the CNES does not specify whether the professional listed as the manager or administrator is also the owner of the establishment, a cross-check was carried out using the platform of the National Network for the Simplification of Registration and Legalization of Companies and Businesses (REDESIM), in the consultation section of the National Register of Legal Entities (CNPJ). Each establishment’s CNPJ number identified in the CNES was consulted in order to check whether the name of the nurse manager or administrator appeared on the company’s list of partners.

### Study variables

The variable in this ecological study was an aggregate measure, in which the data from the establishments was aggregated by year, state and region. The variables extracted were: year of registration, gender of the nurse manager or administrator, number of nurses per establishment, type of establishment, main activity, level of care, legal nature and agreement.

In the trend analysis, the dependent variable was the percentage of establishments (number of establishments per year, divided by the total number of establishments in the period, multiplied by 100). The independent variable was the years.

### Study period

The study was conducted between December 2023 and June 2024. The period of the historical series considered in the data collection was from 2003 to 2023.

### Data processing and analysis

The data were stored in a Microsoft Excel® spreadsheet, excluding inconsistencies after double-checking (duplicate records). The description was presented using absolute (n) and relative (%) frequencies. The number of registered nurses in the establishments was presented by mean and standard deviation. The data was presented by table and map. The choropleth maps were generated using version 3.34.3 of the Qgis software, showing the absolute and relative frequency of establishments by state, using the same calculation as for the dependent variable in the trend analysis.

The study is characterized as a time series because it uses a sequence of data at regular intervals, corresponding to the percentage of establishments per year, from 2003 to 2023. A time series is made up of three components, one of which is trend analysis^(16)^. The Joinpoint regression model was adopted because of its ability to identify one or more changes in the linear trend on a logarithmic scale^(17)^.

The trend analysis was carried out using the Joinpoint Trend Analysis software, version 5.3.0, developed by the National Cancer Institute (Calverton, USA). The following parameters were adopted: the values of the series (percentage of establishments in each year) were defined as the dependent variable (Y), while the years of the period analyzed (2003 to 2023) were considered the independent variable (X). The Confidence Interval (CI) was 95% and the p-value <0.05.

The “Heteroscedastic/correlated errors” option was set to “Standard Error”, and an uncorrelated error model was adjusted. This is the standard selection, which considers that the random errors have non-constant variance (are heteroscedastic) and estimates the regression coefficients using the Weighted Least Squares (WLS) method^(18)^. The most appropriate Joinpoint regression model was chosen using the Monte Carlo permutation test, with 4,499 permutations. This method allows us to identify how much larger a proportion needs to be to be statistically significant^(17)^.

Two indicators were used to describe the trends identified: the Annual Percentage Change (APC), which assumes that the proportion of establishments changes at a constant percentage in relation to the previous year and varies linearly on a logarithmic scale^(19)^; and the Average Annual Percentage Change (AAPC), which is a measure that summarizes the trend in a pre-established fixed interval. This metric makes it possible to describe average APCs over several years using a single value. The AAPC is calculated as a weighted average of the model’s APCs, with weights corresponding to the length of each APC’s interval^(20)^.

Trends were classified as increasing or decreasing when the APC and AAPC values were positive or negative, respectively, and showed statistical significance set at p < 0.05. In the absence of significance (p > 0.05), the trend was classified as stationary^(21)^.

### Ethical aspects

This study was approved by the Research Ethics Committee of the Santa Catarina State University, under opinion number 5.440.353.

## Results

Between 2003 and 2023, 340 nursing establishments were found with active CNES registrations, distributed as follows in the Brazilian macro-regions: 17 (5.0%) in the North, 50 (14.71%) in the Northeast, 31 (9.12%) in the Midwest, 137 (40.29%) in the Southeast and 105 (30.88%) in the South. As for the gender of the nurse manager or administrator, 284 (83.53%) were female and 56 (16.47%) male. Regarding the number of registered nurses per establishment, the average was 1.57, with a standard deviation ± 2.67, with a minimum of 1 and a maximum of 45 nurses.

Table 1 shows the characteristics of the nursing establishments. Regarding the type of establishment, isolated practices are the most common, accounting for 128 (37.64%) of the businesses, followed by specialized clinics (n=82; 24.12%), home care services (n=74; 21.76%) and immunization centers (n=31; 9.12%). As for the main activities carried out by nurses, outpatient consultations stand out (n=197; 57.94%), followed by home care and immunization, with 63 (18.53%) and 46 (13.53%) respectively, as shown in Table 1.

**Table 1 -:** Characteristics of nursing establishments in the period 2003-2023 (n = 340). Brazil, 2024

**Variable**		**Regions of Brazil**					
		North 17 (%)	Northeast 50 (%)	Midwest31 (%)	Southeast 137 (%)	South 105 (%)	Brazil 340 (%)*
**Type of establishment**	Immunization center	0 (0.00)	4 (8.00)	4 (12.90)	20 (14.60)	3 (2.86)	31 (9.12)
	Specialized clinic	4 (23.53)	26 (52.00	12 (38.72)	24 (17.52)	16 (15.24)	82 (24.12)
	Isolated clinic	7 (41.18)	9 (18.00)	9 (29.03)	42 (30.65)	61 (58.10)	128 (37.64)
	Cooperative	1 (5.88)	2 (4.00)	2 (6.45)	1 (0.73)	5 (4.76)	11 (3.24)
	Polyclinic	1 (5.88)	0 (0.00)	0 (0.00)	2 (1.46)	2 (1.90)	5 (1.47)
	Home care service	4 (23.53)	9 (18.00)	4 (12.90)	43 (31.39)	14 (13.33)	74 (21.76)
	Diagnostic and therapy support unit	0 (0.00)	0 (0.00)	0 (0.00)	5 (3.65)	4 (3.81)	9 (2.65)
**Main activity**	Administration	1 (5.88)	2 (4.00)	2 (6.45)	1 (0.73)	5 (4.76)	11 (3.24)
	Health care	3 (17.65)	5 (10.00)	1 (3.23)	4 (2.92)	7 (6.67)	20 (5.88)
	Home care	3 (17.65)	6 (12.00)	3 (9.68)	38 (27.74)	13 (12.38)	63 (18.53)
	Outpatient consultation	9 (52.94)	29 (58.00)	20 (64.51)	66 (48.17)	73 (69.52)	197 (57.94)
	Hospitality	0 (0.00)	0 (0.00)	0 (0.00)	1 (0.73)	2 (1.91)	3 (0.88)
	Immunization	1 (5.88)	8 (16.00)	5 (16.13)	27 (19.71)	5 (4.76)	46 (13.53)
**Level of attention**	Primary care	4 (23.53)	4 (8.00)	3 (9.68)	8 (5.84)	41 (39.05)	60 (17.65)
	Medium complexity	13 (76.47)	46 (92.00)	28 (90.32)	128 (93.43)	63 (60.00)	278 (81.76)
	High complexity	0 (0.00)	0 (0.00)	0 (0.0)	1 (0.73)	1 (0.95)	2 (0.59)
**Legal nature**	Cooperative	1 (5.88)	2 (4.00)	2 (6.45)	1 (0.73)	5 (4.76)	11 (3.24)
	Individual entrepreneur	5 (29.41)	9 (18.00)	5 (16.13)	17 (12.41)	24 (22.86)	60 (17.65)
	Limited company	11 (64.71)	37 (74.00)	24 (77.42)	111 (81.02)	72 (68.57)	255 (75.00)
	Simple limited company	0 (0.00)	1 (2.00)	0 (0.00)	6 (4.38)	4 (3.81)	11 (3.24)
	Pure simple company	0 (0.00)	1 (2.00)	0 (0.00)	2 (1.46)	0 (0.00)	3 (0.87)
**Agreement**	Private	15 (88.24)	25 (50.00)	18 (58.06)	69 (50.36)	69 (65.71)	196 (57.65)
	Private health insurance	2 (11.76)	23 (46.00)	13 (41.94)	64 (46.72)	32 (30.48)	134 (39.41)
	Private and SUS ^†^	0 (0.0)	2 (4.00)	0 (0.00)	4 (2.92)	4 (3.81)	10 (2.94)

Source: National Register of Health Establishments (CNES) *% = Relative Frequency; ^†^SUS = Unified Health System

When analyzing the levels of health care provided, there is a diverse distribution in terms of the complexity of the services offered. Thus, most of the care is related to medium complexity (n=278; 81.76%), followed by basic care (n=60; 17.65%). The presence of high-complexity care is minimal, representing only 0.59% of the enterprises (Table 1). In terms of legal nature, the limited company is predominant in most types of establishments (n=255; 75.00%), followed by the individual entrepreneur (n=60; 17.65%). Other legal categories, such as simple limited companies or pure simple companies, have a less significant presence (Table 1).

Furthermore, when analyzing the types of agreements made available for payment of the services provided, it can be seen that the majority accept private payment exclusively (n= 196; 57.65%). On the other hand, a considerable proportion (n=134; 39.41%) of establishments allow the use of both private health plans and private payment, while a smaller proportion (n=10; 2.94%) provide services through private payment, private health plans and the SUS (Table 1).

**Figure 1:**
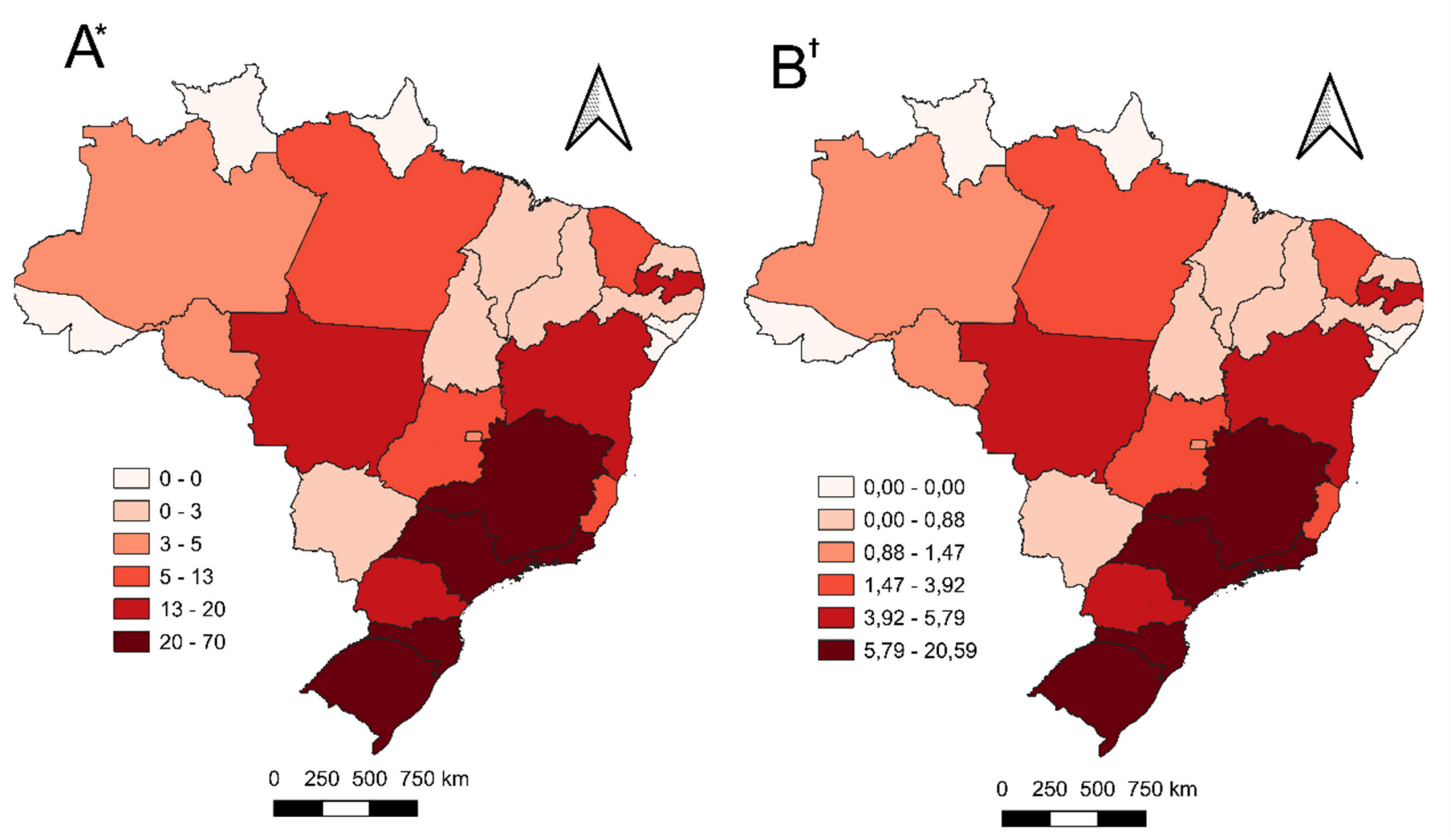
- Spatial distribution of nursing establishments in the federative units between 2003-2023 (n = 340). Brazil, 2024

Figure 1 shows, respectively, maps with the distribution of establishments in the federal units, in terms of absolute frequency (Figure 1 A) and relative frequency (Figure 1 B). In this context, Rio Grande do Sul, Santa Catarina, São Paulo, Minas Gerais and Rio de Janeiro stand out for their wider coverage of autonomous nursing services compared to other areas of the country. These states have a number of establishments ranging from 20 to 70, representing a percentage range of 5.79% to 20.59%.

Also noteworthy are Mato Grosso, Bahia, Paraíba and Paraná, with between 13 and 20 establishments, corresponding to a percentage range between 3.92% and 5.79% (Figure 1 A and Figure 1 B). The Northern region had the lowest percentage of nursing establishments, with only 17, representing 5.0% of the total.

*A= Absolute frequency of nursing establishments; ^†^B = Relative frequency of nursing establishments

Table 2 shows the time distribution of nursing establishments from 2003 to 2023. It can be seen that in 2021 there were the highest number of registered establishments.

**Table 2 -:** Time distribution of nursing establishments, 2003-2023. Brazil, 2024

**Year**	**N** *	**%** ^†^	**Standard error** ^‡^
2003	1	0.29	0.29
2004	1	0.29	0.29
2005	1	0.29	0.29
2006	1	0.29	0.29
2007	2	0.59	0.41
2008	3	0.88	0.50
2009	2	0.59	0.41
2010	2	0.59	0.41
2011	6	1.76	0.71
2012	6	1.76	0.71
2013	6	1.76	0.71
2014	9	2.65	0.87
2015	12	3.53	1.00
2016	13	3.82	1.04
2017	16	4.71	1.14
2018	28	8.24	1.49
2019	31	9.12	1.56
2020	46	13.53	1.85
2021	63	18.53	2.10
2022	47	13.82	1.87
2023	46	13.53	1.85

*N= Absolute frequency; ^†^% = Relative frequency; ^‡^Standard error in each period (year)

Table 3 shows the time trend analysis of entrepreneurship in nursing establishments in Brazil. Over the 20 years of the study, two distinct periods were observed. Between 2003 and 2021, the analysis revealed an upward trend, expressed by the APC of 30.07% per year (95% CI: 28.51-34.65), with a p-value = 0.00. However, from 2021 to 2023, the analysis indicated a negative, but not significant, percentage change of -12.45% (95% CI: -24.09; 1.67; p = 0.17) (Table 3).

**Table 3 -:** Time trend analysis of nursing establishments, 2003-2023. Brazil, 2024

**Period**	APC*	p **- value** ^†^	**95%CI** ^‡^	**Trends**
2003 – 2021	30.07	0.00	28.51 – 34.65	Growing
2021 – 2023	-12.45	0.17	-24.09 – 1.67	Stationary
2003 – 2023	25.01 ^§^	0.00	23.55 – 28.65	Growing

*APC = Annual Percentage Change; ^†^p < 0.05; ^‡^95% CI= 95% Confidence Interval; ^§^AAPC = Average Annual Percentage Change

When analyzing the total period (2003-2023), the AAPC was 25.01% (95% CI: 23.55 - 28.65), with a p-value = 0.00, indicating an upward trend in these ventures over these years, as shown in Table 3.

## Discussion

The results of this study reveal important aspects about the spatial and temporal distribution of nursing care establishments in Brazil, as well as the characterization of the types of establishments, main activities and levels of health care.

With regard to the distribution of establishments in the regions of Brazil, there is a concentration in the Southeast and South. In a study on the distribution of physiotherapists carried out in the CNES, the Southeast region also stood out, with 50%^(22)^. Although the data from this study cannot be considered definitive for providing a complete overview of business entrepreneurship in nursing care establishments, the expressiveness of the Southeast region is not unexpected considering its economic development throughout Brazil’s history and the concentration of places on undergraduate courses^(23)^ and of nursing professionals in the state of São Paulo, over 700,000 of whom 183,000 are nurses^(24)^.

The concentration of Brazil’s productive activity in the southeast region stands out, as does the centralization of political power and economic dominance, which led to a rapid transition from the agro-export model to the industrial model, gaining long-lasting advantages in the process^(25)^. From the perspective of business entrepreneurship, the concentration of Gross Domestic Product (GDP) in the aforementioned regions stands out, as well as their higher per capita income, making them potentially favorable for entrepreneurship because they have a heated consumer market, with the states of São Paulo, Rio de Janeiro, Rio Grande do Sul and Santa Catarina in four of the top five positions^(26)^.

With regard to the number of registrations over the years, the data shows a more expressive growth from 2018 onwards, possibly influenced by changes in the regulatory context of the profession, such as COFEN Resolution 568/2018. Other resolutions, such as COFEN No. 685/2022, which establishes the Technical Responsibility Annotation (ART) in autonomous and/or liberal nursing services, may also be related to this increase. In this context, it is also pertinent to mention that, in a period similar to the data collection, 2005 to 2021, a study identified that COFEN published 385 resolutions that support nursing practice^(27)^.

However, there is no evidence to establish a direct causal relationship between these regulations and the increase observed in the data. Other factors, such as the Gross Domestic Product (GDP) and the Human Development Index (HDI), may also have influenced this growth in the period analyzed. Thus, future research is needed to evaluate not only the effectiveness of COFEN’s resolutions, but also the role of these factors in fostering business entrepreneurship in nursing, enabling a broader understanding of the determinants of the growth of these establishments in Brazil.

Although the data suggests growth, it may not reflect even a small proportion of nursing establishments, since there is no consolidated overview of this scenario in the CORENs. Furthermore, registration with the CNES is not compulsory for establishments to register with the regional nursing councils. However, it is a requirement for agreements with private plans, which account for a significant 39.41% of the data. Although nurses as liberal professionals can only work with their Individual Taxpayer Registration (CPF), there is a movement to professionalize management and seek out new spaces for which formalization, such as with the CNES, is necessary.

The predominant types of nursing establishments in Brazil, according to CNES data, are isolated practices, specialized clinics and home care services. The main activities include outpatient consultations, home care and immunization, demonstrating a focus on direct and preventive care actions. However, most of these establishments operate in secondary health care, focusing on medium-complexity services. As for payment, most are paid directly according to the services provided or through health plans.

Internationally, there is a greater emphasis on primary care^(28)^, especially in activities such as private practice and home care^(29-31)^. In Canada, self-employed nurses excel in specialized services such as wound care and wellness consulting^(32)^. In Switzerland, self-employed nursing practice is regulated, allowing for work in various specialties. In 2016, the Swiss Nurses Association (ASE) registered around 1,800 professionals in this field^(33)^. By October 2024, in the canton of Ticino, located in the south of the country, 199 nurses were working in areas such as home care, wound care, podiatry, cancer and palliative care, obstetrics, breastfeeding and geriatrics, offering everything from basic care to specialized services, with options for 24-hour care or scheduled appointments^(34)^.

In Italy, regulations guarantee nurses full autonomy to organize and carry out their activities autonomously, allowing both individual practices and the formation of associations and cooperatives^(35)^. The system also provides specific social security coverage for self-employed nurses, strengthening professional recognition and encouraging entrepreneurship^(36)^. In addition, a platform developed by the National Federation of Nursing Professions connects citizens to self-employed nurses, facilitating access to services such as home care, medication administration and wound care, promoting visibility and appreciation of the category^(37)^.

In Brazil, COFEN Resolution No. 568/2018, which authorizes the operation of nursing clinics and practices, represents an important regulatory milestone for expanding entrepreneurial opportunities in the profession. This regulation finds parallels in other countries, such as the Full Practice Authority (FPA) implemented in the United States, which grants nurse specialists autonomy to diagnose, initiate and manage treatments, including prescribing medications and controlled substances^(38)^. In the USA, states that have adopted FPA, there has been a 374.1% increase in the likelihood of autonomous practice, as well as a greater presence of these professionals in areas with a shortage of primary health care^(39)^.

In the Philippines, the EntrepreNurse project, launched in 2010, exemplifies the impact of public policies in fostering entrepreneurship. The program encouraged nurses to set up their own businesses, especially in regions with a shortage of professionals, by providing funding, business training and contractual support. As a result, nurses opened home care businesses and rehabilitation clinics^(40)^.

Similarly, in Thailand, the National Health Security Office (NHSO) implemented a health policy in 2019 that established more than 100 nursing and midwifery clinics to expand access to primary services in rural communities, reducing hospital overload and strengthening nurses’ autonomy as leaders and managers of health services^(41)^.

Although the regulatory contexts vary, all these initiatives demonstrate how regulatory changes broaden the scope of practice of nurses, foster entrepreneurship and improve access to healthcare. However, the development of entrepreneurship in nursing faces challenges, such as health policies, funding, educational systems, gender roles, geographical location, as well as ethical-legal barriers, such as legislation specific to each state or country, which can either limit or expand opportunities for autonomous practice in nursing^(42-45)^.

Globally, entrepreneurship in nursing is still not very significant, covering only 0.5% to 1% of professionals^(46)^. However, the potential for expansion is significant, especially with the inclusion of entrepreneurship content in nursing curricula, enabling professionals to overcome structural and cultural barriers and develop innovative and sustainable care models^(47)^. Using their skills and experience, entrepreneurial nurses can take on leadership roles, but this area still requires greater scientific grounding and presence in nursing education curricula^(41)^.

Entrepreneurship can also be a way of solving problems of the past and possibilities for a sustainable future for services and people, in particular the new generation of nursing professionals, in relation to Sustainable Development Goal 8, to promote sustained, inclusive and sustainable economic growth, full and productive employment and decent work for all^(48)^. In this sense, sustainability through entrepreneurship not only has the potential to meet the basic health demands of the population, but also to produce wealth and jobs.

The Pan American Health Organization (PAHO) definition highlights primary health care (PHC) as the first point of contact capable of meeting up to 90% of a person’s health needs, ranging from promotion and prevention (such as vaccinations) to treatment, palliative care and rehabilitation^(49)^. By comparing this definition with the types of establishments and main activities described in the CNES, it is possible to establish a clear relationship between PHC and business entrepreneurship in nursing.

This study has some limitations that need to be considered. Firstly, the analysis was based on a single database, which may limit the scope of the results. In addition, the scarcity of similar studies in the nursing field makes it difficult to compare and contextualize the findings, reinforcing the need to expand research on the subject at national and international level.

Another limitation refers to the impossibility of confirming, in the case of establishments registered as Individual Entrepreneurs, whether the nurse listed as manager or administrator in the CNES is also the owner of the business. This legal structure has no corporate structure, linking the CNPJ directly to the owner of the company. This makes verification through REDESIM impossible and makes it difficult to distinguish between nurses who are owners and those hired to manage the establishment.

This issue highlights the importance of future research that adopts analysis methods at the individual level, allowing for more precise identification of business ownership and a more robust understanding of the entrepreneurial activity of nurses in Brazil. In addition, it is necessary to investigate whether the types of establishments, main activities and level of care are really concentrated in medium complexity, as the data analyzed suggests.

Despite these limitations, this study stands out as the first, according to the available literature, to characterize and describe the spatial and temporal distribution of nursing care establishments at a national level in Brazil. The results offer unprecedented data on business entrepreneurship in nursing, broadening the understanding of the subject and providing a solid basis for future research.

## Conclusion

There has been a significant increase in registrations, especially following the publication of COFEN resolutions that encourage autonomous and liberal practice in nursing, broadening the scope of nurses’ work and promoting opportunities for entrepreneurship in the area. Formalization and registration with the CNES represent a step forward in the professionalization of nursing management, and are essential for securing benefits such as agreements with health plans, professional recognition and tax incentives. Only with formal registration and a consolidated panorama is it possible to strengthen the profession and create a favorable environment for the sustainable development of the sector.
