# Supplementary material for: Business entrepreneurship in nursing care facilities in Brazil: an ecological study
Source: Rev Lat Am Enfermagem. 2025 Jul 11;33:e4584. doi: 10.1590/1518-8345.7657.4584 (PMC12259216; doi:10.1590/1518-8345.7657.4584)
Supplement: Translation

# Translation: Empreendedorismo de negócios em estabelecimentos assistenciais de enfermagem no Brasil: um estudo ecológico

**DOI:** 10.1590/1518-8345.7657.4585

**Authors:** Matheus Moraes Silva, Jouhanna do Carmo Menegaz, Glenda Roberta Oliveira Naiff Ferreira, Josele de Jesus Quaresma Trindade Costa, Maria Eduarda Libório Martins, Allison Squires

**Affiliations:** 1 Universidade Federal do Pará, Faculdade de Enfermagem, Belém, PA, Brasil.; 2 Bolsista da Coordenação de Aperfeiçoamento de Pessoal de Nível Superior (CAPES), Brasil.; 3 Universidade do Estado de Santa Catarina, Departamento de Enfermagem, Chapecó, SC, Brasil.; 4 Bolsista do Conselho Nacional de Desenvolvimento Científico e Tecnológico (CNPq), Brasil.; 5 New York University, Rory Meyers College of Nursing, New York, NY, Estados Unidos da América.

**Keywords:** Empreendedorismo, Serviços de Enfermagem, Mercado de Trabalho, Prática Privada de Enfermagem, Autonomia Profissional, Acesso e Cobertura Universal de Saúde

## Abstract

caracterizar e descrever a distribuição espacial e temporal dos estabelecimentos assistenciais de enfermagem no Brasil segundo o Cadastro Nacional de Estabelecimentos de Saúde.

estudo ecológico, utilizando dados secundários do Cadastro Nacional de Estabelecimentos de Saúde, agregados por ano, estado e região. As variáveis extraídas foram: ano de cadastro, sexo do enfermeiro gerente ou administrador, número de enfermeiros por estabelecimento, tipo de estabelecimento, atividade principal, nível de atenção, natureza jurídica e convênio. Realizou-se estatística descritiva e análise temporal no programa *Jointpoint*.

entre 2003 e 2023, foram encontrados 340 estabelecimentos de enfermagem com cadastro ativo. As regiões Sul e Sudeste concentraram a maior parte dos estabelecimentos. Houve predomínio de consultórios, clínicas especializadas e serviços de atenção domiciliar. A análise temporal indicou que, entre 2003 e 2021, houve um crescimento anual de 30,07% (*p*= 0,00). Considerando o período total (2003-2023), houve um crescimento anual de 25,01%, indicando tendência crescente dos empreendimentos ao longo dos anos.

a formalização e o registro refletem a profissionalização da gestão em enfermagem. Observa-se um crescimento nos cadastros após a publicação de resoluções do Conselho Federal de Enfermagem que apoiam a prática autônoma e/ou liberal.

## Destaques

**(1)** As regiões Sul e Sudeste concentram mais estabelecimentos de enfermagem. **(2)** Entre 2003 e 2023, houve um crescimento anual de 25,01% nos empreendimentos. **(3)** Predominam consultórios isolados e clínicas especializadas. **(4)** As atividades principais são: consulta ambulatorial, atenção domiciliar e imunização. **(5)** A maioria aceita pagamento particular e planos de saúde privados.

## Introdução

O empreendedorismo na enfermagem é definido como a habilidade de identificar oportunidades, desenvolver projetos inovadores e implementar soluções para a melhoria contínua do cuidado em saúde. O conceito vai além da criação de empresas, abrangendo inovação, pesquisa e avanço científico, permitindo que os enfermeiros transformem práticas de saúde e atendam às demandas da sociedade^(1)^.

No cenário internacional, o empreendedorismo em enfermagem tem sido amplamente reconhecido por seu potencial de enfrentar desafios complexos e promover inovações na prestação de serviços de saúde^(2)^. Uma revisão sistemática destacou que enfermeiros empreendedores identificam lacunas nos sistemas de saúde e desenvolvem serviços inovadores, como clínicas especializadas, empresas de atendimento domiciliar e consultórios independentes, diversificando os serviços e ampliando o acesso aos cuidados^(3)^.

Em 2023, a *American Nurses Association Enterprise Innovation* destacou que mais de 300 enfermeiros empreendedores lideraram iniciativas como agentes de mudança. Essas ações incluíram o desenvolvimento de dispositivos médicos, novos modelos na prestação de cuidados, criação de negócios, pesquisa e especialidades educacionais, evidenciando o papel do empreendedorismo no avanço da profissão^(4)^.

De modo similar, no Brasil, o setor de saúde está preparado para uma expansão impulsionada por mudanças demográficas, políticas e transformações nas atitudes dos consumidores. O envelhecimento da população, o aumento das doenças crônicas e o crescimento da classe média têm gerado uma demanda crescente por serviços e tecnologias de saúde avançadas^(5-7)^. Além disso, o crescente interesse dos brasileiros pela saúde preventiva está impulsionando o desenvolvimento de subsetores, como clínicas de saúde e bem-estar^(8)^.

Logo, esse contexto destaca oportunidades empreendedoras no campo da enfermagem. O direito de empreender e ter o próprio negócio na enfermagem é respaldado pela Lei do Exercício Profissional nº 7.498/1986, que assegura a autonomia do enfermeiro. Ademais, o empreendedorismo de negócios na enfermagem destaca-se no cenário brasileiro, sobretudo com a Resolução nº 568/2018, do Conselho Federal de Enfermagem (COFEN), que regulamenta a atuação de enfermeiros em clínicas e consultórios de enfermagem^(9)^.

Assim, para os propósitos da resolução supracitada, a clínica de enfermagem é definida como um estabelecimento constituído por consultórios e ambientes destinados ao atendimento de enfermagem individual, coletivo e/ou domiciliar. Por outro lado, o consultório de enfermagem é definido como a área física exclusiva para consultas e outras atividades privativas do enfermeiro^(9)^. Tais definições reforçam o direito de empreender e exercer a profissão de enfermagem de maneira autônoma e independente.

O empreendedor de negócios em enfermagem que possui um estabelecimento assistencial, entendido neste estudo como clínicas ou consultórios, necessita atender a uma série de requisitos jurídicos, tributários, sanitários e regulamentares perante o Conselho Regional de Enfermagem para poder atuar de forma ética e legal^(10)^. Nesse sentido, os estabelecimentos assistenciais são espaços físicos delimitados e permanentes no qual são realizados ações e serviços de saúde humana sob responsabilidade técnica^(11)^.

No contexto brasileiro, a gestão dos estabelecimentos assistenciais é normatizada pelo Cadastro Nacional de Estabelecimentos de Saúde (CNES), um sistema informatizado mantido pelo Ministério da Saúde que centraliza informações de todos os serviços de saúde no país, sejam eles públicos ou privados, com ou sem convênio com o Sistema Único de Saúde (SUS). Vale ainda ressaltar que é obrigatório que todos os estabelecimentos que ofereçam atendimento à saúde humana possuam um registro^(11)^.

Considerando o pouco conhecimento sobre o empreendedorismo de negócios em estabelecimentos assistenciais de enfermagem, a escassez de estudos que mapeiem suas características e tendências temporais no Brasil, bem como a ausência de dados unificados disponibilizados pelos Conselhos Regionais de Enfermagem (CORENs) e pelo COFEN sobre o panorama desses empreendimentos, este estudo teve como objetivo caracterizar e descrever a distribuição espacial e temporal dos estabelecimentos assistenciais de enfermagem no Brasil, segundo o CNES.

## Método

### Delineamento do estudo

Trata-se de um estudo ecológico, guiado pela ferramenta *Strengthening the Reporting of Observational Studies in Epidemiology* (STROBE). As análises e os resultados de estudos ecológicos são aplicáveis apenas no nível populacional^(12)^. Neste estudo, as unidades de análise foram os 27 estados e as 5 (cinco) regiões administrativas do Brasil.

### Cenário

O estudo foi conduzido no território brasileiro, que possui uma extensão territorial de 8.510.417,77 km², dividida em 26 estados, um Distrito Federal e 5.570 municípios. O país está organizado em cinco macrorregiões: Norte, Nordeste, Centro-Oeste, Sudeste e Sul. Até agosto de 2022, a população brasileira era estimada em 203.080.756 indivíduos^(13)^.

Segundo dados do COFEN, o país possui cerca de 756.054 profissionais enfermeiros registrados até novembro de 2024^(14)^. Quanto à proporção de enfermeiros por 1.000 habitantes nas macrorregiões do Brasil, um estudo realizado em 2021, baseado em dados do perfil da enfermagem publicado pelo COFEN em 2016, apresentou as seguintes estatísticas: Região Norte com 1,6 por 1.000 habitantes, Região Nordeste com 1,76 por 1.000 habitantes, Região Centro-Oeste com 2,25 por 1.000 habitantes, Região Sudeste com 2,38 por 1.000 habitantes e Região Sul com 1,77 por 1.000 habitantes^(15)^.

### População e critérios de seleção

Foram incluídos os dados dos estabelecimentos assistenciais de enfermagem classificados como Entidades Empresariais e Pessoas Físicas (grupo), nos quais o profissional enfermeiro atuava como gerente ou administrador, considerando apenas aqueles com cadastro ativo no CNES. Foram excluídos os dados que apresentavam informações incompletas ou não atendiam aos requisitos pré-estabelecidos.

Destaca-se ainda que não foi definido nenhum recorte temporal para a seleção dos dados, visando integrar o maior número de estabelecimentos, a fim de dimensionar adequadamente a evolução longitudinal do empreendedorismo em estabelecimentos assistenciais de enfermagem ao longo do tempo.

### Coleta de dados

Para a coleta de dados, utilizou-se a plataforma do CNES, na seção de Consulta de Estabelecimento, empregando as palavras-chave: “Clínica de Enfermagem”, “Consultório de Enfermagem”, “Enfermeiro”, “Enfermeira”, “Nursing” e “Nurse”. Essa metodologia de busca se justifica pela ausência de uma opção que permita pesquisar os estabelecimentos por categoria profissional de saúde. Dessa forma, optou-se pela estratégia supracitada com a finalidade de filtrar os estabelecimentos relacionados à enfermagem pelo nome fantasia ou empresarial.

Como o CNES não especifica se o profissional listado como gerente ou administrador também é proprietário do estabelecimento, foi realizada uma verificação cruzada, utilizando a plataforma da Rede Nacional para a Simplificação do Registro e da Legalização de Empresas e Negócios (REDESIM), na seção consulta de Cadastro Nacional de Pessoa Jurídica (CNPJ). Cada número de CNPJ dos estabelecimentos identificado no CNES foi consultado, com o objetivo de verificar se o nome do enfermeiro gerente ou administrador constava no quadro de sócios da empresa.

### Variáveis do estudo

A variável deste estudo ecológico foi uma medida agregada, na qual os dados dos estabelecimentos foram agregados por ano, estado e região. As variáveis extraídas foram: ano de cadastro, sexo do enfermeiro gerente ou administrador, número de enfermeiros por estabelecimento, tipo de estabelecimento, atividade principal, nível de atenção, natureza jurídica e convênio.

Em contrapartida, na análise de tendência, a variável dependente foi o percentual de estabelecimentos (número de estabelecimentos por ano, divididos pelo total de estabelecimentos no período e multiplicado por 100). A variável independente foram os anos.

### Período do estudo

O estudo foi conduzido entre os meses de dezembro de 2023 e junho de 2024. O período da série histórica considerada na coleta de dados foi de 2003 e 2023.

### Tratamento e análise dos dados

Os dados foram armazenados em uma planilha do *Microsoft Excel*®, excluindo inconsistências após a dupla verificação (duplicidade de registro). Já a descrição foi apresentada usando frequências absolutas (n) e relativas (%). O número de enfermeiros registrados nos estabelecimentos foi apresentado por média e desvio-padrão. Além do mais, os dados foram apresentados por tabela e mapa. Por sua vez, os mapas coropléticos foram gerados usando a versão 3.34.3 do *software* Qgis, apresentando a frequência absoluta e relativa dos estabelecimentos por estado, utilizando o mesmo cálculo realizado na variável dependente da análise de tendência.

Ademais, o estudo é caracterizado como uma série temporal por utilizar uma sequência de dados em intervalos regulares, correspondente ao percentual de estabelecimentos por ano, no período de 2003 a 2023. Uma série histórica é composta por três componentes, sendo a análise de tendência um deles^(16)^. Inclusive, o modelo de regressão do *Joinpoint* foi adotado por sua capacidade de identificar uma ou mais mudanças na tendência linear em escala logarítmica^(17)^.

A análise de tendência foi realizada utilizando o *software Joinpoint Trend Analysis*, versão 5.3.0, desenvolvido pelo *National Cancer Institute* (Calverton, Estados Unidos da América). Foram adotados os seguintes parâmetros: os valores da série (percentual de estabelecimentos em cada ano) foram definidos como variável dependente (Y), enquanto os anos do período analisado (2003 a 2023) foram considerados como variável independente (X). Sendo assim, o Intervalo de Confiança (IC) foi de 95% e o valor de *p* <0,05.

A opção “Erros heterocedásticos/correlacionados” foi configurada como “Erro-Padrão”, e um modelo de erros não correlacionados foi ajustado. Essa é a seleção padrão, que considera que os erros aleatórios possuem variância não constante (são heterocedásticos) e estima os coeficientes de regressão por meio do método de mínimos quadrados ponderados (*Weighted Least Squares* – WLS)^(18)^. A escolha do modelo de regressão *Joinpoint* mais adequado foi feita por meio do teste de permutação Monte Carlo, com 4.499 permutações. Esse método permite identificar o quanto uma proporção precisa ser maior para ser estatisticamente significativa^(17)^.

Além do mais, foram utilizados dois indicadores para descrever as tendências identificadas: a Variação Percentual Anual (*Annual Percent Change* – APC), que pressupõe que a proporção de estabelecimentos muda a uma porcentagem constante em relação ao ano anterior e varia linearmente em escala logarítmica^(19)^; e a Variação Percentual Anual Média (*Average Annual Percentage Change* – AAPC), que é uma medida que resume a tendência em um intervalo fixo preestabelecido. Tal métrica permite descrever os APCs médios ao longo de vários anos usando um único valor. Logo, o cálculo da AAPC é realizado como uma média ponderada dos APCs do modelo, com pesos correspondentes ao comprimento do intervalo de cada APC^(20)^.

Em suma, as tendências foram classificadas como crescentes ou decrescentes quando os valores de APC e AAPC eram positivos ou negativos, respectivamente, e apresentavam significância estatística estabelecida em p < 0,05. Na ausência de significância (p > 0,05), a tendência foi classificada como estacionária^(21)^.

### Aspectos éticos

Este estudo possui aprovação no Comitê de Ética e Pesquisa da Universidade do Estado de Santa Catarina, sob o parecer número 5.440.353.

## Resultados

Entre os anos de 2003 e 2023, foram encontrados 340 estabelecimentos de enfermagem com cadastro ativo no CNES, distribuídos da seguinte forma nas macrorregiões brasileiras: 17 (5,0%) no Norte, 50 (14,71%) no Nordeste, 31 (9,12%) no Centro-Oeste, 137 (40,29%) no Sudeste e 105 (30,88%) no Sul. Quanto ao sexo do enfermeiro gerente ou administrador, 284 (83,53%) era do sexo feminino e 56 (16,47%) do sexo masculino. Em relação ao número de enfermeiros registrados por estabelecimento, a média foi de 1,57, com um desvio-padrão ± 2,67, com o mínimo de 1 (um) e o máximo de 45 enfermeiros.

Na Tabela 1 são apresentadas as características dos estabelecimentos de enfermagem. No que diz respeito ao tipo de estabelecimento, os consultórios isolados são os mais comuns, representando 128 (37,64%) dos empreendimentos, seguidos pelas clínicas especializadas (n=82; 24,12%), serviços de atenção domiciliar (n=74; 21,76%) e centros de imunização (n=31; 9,12%). Quanto às principais atividades desenvolvidas pelos enfermeiros, destaca-se a consulta ambulatorial (n=197;57,94%), seguida pela atenção domiciliar e pela imunização, com respectivamente 63 (18,53%) e 46 (13,53%), conforme também apresentado na Tabela 1.

**Tabela 1 -:** Características dos estabelecimentos de enfermagem, no período de 2003-2023 (n = 340). Brasil, 2024

**Variável**		**Regiões do Brasil**					
		Norte 17 (%)	Nordeste 50 (%)	Centro-oeste 31 (%)	Sudeste 137 (%)	Sul 105 (%)	Brasil 340 (%)*
**Tipo de estabelecimento**	Centro de imunização	0 (0,00)	4 (8,00)	4 (12,90)	20 (14,60)	3 (2,86)	31 (9,12)
	Clínica especializada	4 (23,53)	26 (52,00	12 (38,72)	24 (17,52)	16 (15,24)	82 (24,12)
	Consultório isolado	7 (41,18)	9 (18,00)	9 (29,03)	42 (30,65)	61 (58,10)	128 (37,64)
	Cooperativa	1 (5,88)	2 (4,00)	2 (6,45)	1 (0,73)	5 (4,76)	11 (3,24)
	Policlínica	1 (5,88)	0 (0,00)	0 (0,00)	2 (1,46)	2 (1,90)	5 (1,47)
	Serviço de atenção domiciliar ( *Home care* )	4 (23,53)	9 (18,00)	4 (12,90)	43 (31,39)	14 (13,33)	74 (21,76)
	Unidade de apoio, diagnóstico e terapia	0 (0,00)	0 (0,00)	0 (0,00)	5 (3,65)	4 (3,81)	9 (2,65)
**Atividade principal**	Administração	1 (5,88)	2 (4,00)	2 (6,45)	1 (0,73)	5 (4,76)	11 (3,24)
	Assistência à saúde	3 (17,65)	5 (10,00)	1 (3,23)	4 (2,92)	7 (6,67)	20 (5,88)
	Atenção domiciliar	3 (17,65)	6 (12,00)	3 (9,68)	38 (27,74)	13 (12,38)	63 (18,53)
	Consulta ambulatorial	9 (52,94)	29 (58,00)	20 (64,51)	66 (48,17)	73 (69,52)	197 (57,94)
	Hospitalidade	0 (0,00)	0 (0,00)	0 (0,00)	1 (0,73)	2 (1,91)	3 (0,88)
	Imunização	1 (5,88)	8 (16,00)	5 (16,13)	27 (19,71)	5 (4,76)	46 (13,53)
**Nível de atenção**	Atenção básica	4 (23,53)	4 (8,00)	3 (9,68)	8 (5,84)	41 (39,05)	60 (17,65)
	Média complexidade	13 (76,47)	46 (92,00)	28 (90,32)	128 (93,43)	63 (60,00)	278 (81,76)
	Alta complexidade	0 (0,00)	0 (0,00)	0 (0,0)	1 (0,73)	1 (0,95)	2 (0,59)
**Natureza jurídica**	Cooperativa	1 (5,88)	2 (4,00)	2 (6,45)	1 (0,73)	5 (4,76)	11 (3,24)
	Empresário Individual	5 (29,41)	9 (18,00)	5 (16,13)	17 (12,41)	24 (22,86)	60 (17,65)
	Sociedade empresária limitada	11 (64,71)	37 (74,00)	24 (77,42)	111 (81,02)	72 (68,57)	255 (75,00)
	Sociedade simples limitada	0 (0,00)	1 (2,00)	0 (0,00)	6 (4,38)	4 (3,81)	11 (3,24)
	Sociedade simples pura	0 (0,00)	1 (2,00)	0 (0,00)	2 (1,46)	0 (0,00)	3 (0,87)
	Particular	15 (88,24)	25 (50,00)	18 (58,06)	69 (50,36)	69 (65,71)	196 (57,65)
**Convênio**	Plano de saúde privado e particular	2 (11,76)	23 (46,00)	13 (41,94)	64 (46,72)	32 (30,48)	134 (39,41)
	Particular, SUS ^†^ e plano de saúde privado.	0 (0,0)	2 (4,00)	0 (0,00)	4 (2,92)	4 (3,81)	10 (2,94)

Fonte: Cadastro Nacional de Estabelecimentos de Saúde (CNES) *% = Frequência Relativa; ^†^SUS = Sistema Único de Saúde

Ao analisar os níveis de atenção prestados à saúde, verifica-se uma distribuição diversificada em termos de complexidade dos serviços oferecidos. Assim, a maioria dos atendimentos está relacionada à média complexidade (n=278; 81,76%), seguida pela atenção básica (n=60; 17,65%). A presença de atendimentos de alta complexidade é mínima, representando apenas 0,59% dos empreendimentos (Tabela 1). Em relação à natureza jurídica, a sociedade empresária limitada é predominante na maioria dos tipos de estabelecimentos (n=255; 75,00%), seguida pelo empresário individual (n=60; 17,65%). Outras categorias jurídicas, como sociedades simples limitada ou sociedades simples puras, têm uma presença menos expressiva (Tabela 1).

Além disso, ao analisar os tipos de convênios disponibilizados para o pagamento dos serviços prestados, observa-se que a maioria aceita exclusivamente pagamento particular (n= 196; 57,65%). Por outro lado, uma parcela considerável (n=134; 39,41%) dos estabelecimentos permite o uso tanto de planos de saúde privados quanto o pagamento particular, enquanto uma menor proporção (n=10; 2,94%) atende por meio de pagamento particular, de plano de saúde privado e pelo SUS (Tabela 1).

Na Figura 1 são apresentados, respectivamente, os mapas com a distribuição dos estabelecimentos nas unidades federativas, em termos de frequência absoluta (Figura 1) e relativa (Figura 1). Nesse contexto, destacam-se os estados do Rio Grande do Sul, Santa Catarina, São Paulo, Minas Gerais e Rio de Janeiro pela cobertura mais ampla de serviços autônomos de enfermagem em comparação com outras áreas do país. Esses estados possuem um número de estabelecimentos que varia de 20 a 70, representando uma faixa percentual de 5,79% a 20,59%.

Além disso, também merecem destaque o Mato Grosso, a Bahia, a Paraíba e o Paraná, com um quantitativo entre 13 e 20 estabelecimentos, o que corresponde a uma faixa percentual entre 3,92% e 5,79% (Figura 1A e Figura 1B). A região Norte apresentou o menor percentual de estabelecimentos de enfermagem, com apenas 17, representando 5,0% do valor total.

**Figura 1 -:** Distribuição espacial dos estabelecimentos de enfermagem nas unidades federativas, entre 2003-2023 (n = 340). Brasil, 2024

*A= Frequência absoluta de estabelecimentos de enfermagem; ^†^B = Frequência relativa de estabelecimentos de enfermagem

A Tabela 2 apresenta a distribuição temporal dos estabelecimentos de enfermagem, no período de 2003 a 2023. Observa-se que no ano de 2021 houve o maior número de estabelecimentos registrados.

**Tabela 2 -:** Distribuição temporal de estabelecimentos de enfermagem, 2003-2023. Brasil, 2024

**Ano**	**N** *	**%** ^†^	**Erro-padrão** ^‡^
2003	1	0,29	0,29
2004	1	0,29	0,29
2005	1	0,29	0,29
2006	1	0,29	0,29
2007	2	0,59	0,41
2008	3	0,88	0,50
2009	2	0,59	0,41
2010	2	0,59	0,41
2011	6	1,76	0,71
2012	6	1,76	0,71
2013	6	1,76	0,71
2014	9	2,65	0,87
2015	12	3,53	1,00
2016	13	3,82	1,04
2017	16	4,71	1,14
2018	28	8,24	1,49
2019	31	9,12	1,56
2020	46	13,53	1,85
2021	63	18,53	2,10
2022	47	13,82	1,87
2023	46	13,53	1,85

*N= Frequência absoluta; ^†^% = Frequência relativa; ^‡^Erro-padrão em cada período (ano)

Já a Tabela 3 apresenta a análise de tendência temporal do empreendedorismo em estabelecimentos de enfermagem no Brasil. Durante os 20 anos de estudo, observaram-se dois períodos distintos. Entre 2003 e 2021, a análise revelou uma tendência crescente, expressa pela APC de 30,07% ao ano (IC 95%: 28,51-34,65), com um valor de p = 0,00. Entretanto, de 2021 a 2023, a análise indicou uma variação percentual negativa, mas não foi significativa, de -12,45% (IC 95%: -24,09; 1,67; *p* = 0,17) (Tabela 3).

**Tabela 3 -:** Análise de tendência temporal dos estabelecimentos de enfermagem, 2003-2023. Brasil, 2024

**Período**	APC*	***p* - valor** ^†^	**IC 95%** ^‡^	**Tendência**
2003 – 2021	30,07	0,00	28,51 – 34,65	Crescente
2021 – 2023	-12,45	0,17	-24,09 – 1,67	Estacionária
2003 – 2023	25,01 ^§^	0,00	23,55 – 28,65	Crescente

*APC = Variação Percentual Anual; ^†^ *p* < 0,05; ^‡^IC 95% = Intervalo de Confiança de 95%; ^§^AAPC = Variação Percentual Média Anual

Ao analisar o período total (2003-2023), a AAPC foi de 25,01% (IC 95%: 23,55 – 28,65), com um *p*-valor = 0,00, indicando uma tendência crescente desses empreendimentos ao longo desses anos, conforme demonstrado na Tabela 3.

## Discussão

Os resultados deste estudo revelam importantes aspectos sobre a distribuição espacial e temporal dos estabelecimentos assistenciais de enfermagem no Brasil, assim como a caracterização dos tipos de estabelecimentos, atividades principais e níveis de atenção à saúde.

Quanto à distribuição dos estabelecimentos nas regiões do Brasil, observa-se a concentração no Sudeste e Sul. Em um estudo sobre a distribuição de fisioterapeutas realizado no CNES, a região Sudeste também se destacou, com 50%^(22)^. Ainda que os dados deste estudo não possam ser considerados definitivos para o fornecimento de um panorama completo sobre o empreendedorismo de negócios em estabelecimentos assistenciais de enfermagem, não é inesperada a expressividade da região sudeste, isso porque considera-se o seu desenvolvimento econômico ao longo da história do Brasil, a concentração de vagas em cursos de graduação^(23)^ e, consequentemente, a quantidade de profissionais de enfermagem no estado de São Paulo, que é acima de 700 mil, sendo destes 183 mil enfermeiros^(24)^.

Ainda, destaca-se a concentração da atividade produtiva brasileira na região sudeste, bem como a centralização do poder político e o domínio econômico, levando-a para uma rápida transição do modelo agroexportador para o industrial, obtendo vantagens longevas no processo^(25)^. Da perspectiva que interessa ao empreendedorismo de negócios, destaca-se a concentração do Produto Interno Bruto (PIB) nas regiões mencionadas, bem como as maiores rendas per capita, tornando-as potencialmente favoráveis ao empreendedorismo por possuir mercado consumidor aquecido, com os estados de São Paulo, Rio de Janeiro, Rio Grande do Sul e Santa Catarina ocupando quatro das cinco primeiras posições^(26)^.

No que diz respeito ao quantitativo de cadastros ao longo dos anos, os dados mostram um crescimento mais expressivo a partir de 2018, possivelmente influenciado por mudanças no contexto normativo da profissão, como a Resolução COFEN nº 568/2018. Outras resoluções, como a COFEN nº 685/2022, que institui a Anotação de Responsabilidade Técnica (ART) nos serviços de enfermagem autônomos e/ou liberais, também podem estar relacionadas a esse aumento. Nesse contexto, é também pertinente mencionar que em período similar a coleta de dados, ou seja, 2005 a 2021, um estudo identificou que o COFEN publicou 385 resoluções que respaldam a prática de enfermagem^(27)^.

Contudo, não há evidências que permitam estabelecer uma relação causal direta entre essas normativas e o aumento observado nos dados. Outros fatores, como o Produto Interno Bruto (PIB) e o Índice de Desenvolvimento Humano (IDH), podem ter igualmente influenciado esse crescimento no período analisado. Assim, pesquisas futuras são necessárias para avaliar não apenas a eficácia das resoluções do COFEN, mas também o papel desses fatores no fomento do empreendedorismo de negócios na enfermagem, possibilitando uma compreensão mais ampla dos determinantes do crescimento desses estabelecimentos no Brasil.

Embora os dados sugiram crescimento, é possível que não reflitam nem uma pequena parcela dos estabelecimentos de enfermagem, dado que não há um panorama consolidado nos CORENs sobre esse cenário. Além disso, o registro no CNES não é obrigatório para a inscrição de estabelecimentos nos conselhos regionais de enfermagem. Entretanto, é um requisito para convênios com planos privados, que representam expressivos 39,41% dos dados. Ainda que o enfermeiro como profissional liberal possa atuar somente com o Cadastro de Pessoa Física (CPF), há um movimento de profissionalização da gestão e de buscar novos espaços para os quais a formalização faz-se necessária, tal como no CNES.

Os tipos de estabelecimentos de enfermagem predominantes no Brasil, de acordo com os dados do CNES, são consultórios isolados, clínicas especializadas e serviços de atenção domiciliar (*Home care*). As principais atividades incluem consultas ambulatoriais, atenção domiciliar e imunização, demonstrando um foco em ações de cuidado direto e preventivo. Contudo, a maioria desses estabelecimentos atua na atenção secundária à saúde, voltados para serviços de média complexidade. Quanto à forma de pagamento, a maioria é direto, de acordo com os serviços prestados, ou através de planos de saúde.

No cenário internacional, observa-se maior ênfase nos cuidados primários^(28)^, especialmente em atividades como prática clínica privada e *Home care* ^(29-31)^. No Canadá, enfermeiros autônomos destacam-se em serviços especializados, como cuidados com feridas e consultoria em bem-estar^(32)^. Na Suíça, a prática autônoma na enfermagem é regulamentada, permitindo atuação em diversas especialidades. Em 2016, a Associação Suíça de Enfermeiros (ASE) registrou cerca 1.800 profissionais nessa modalidade^(33)^. Até outubro de 2024, no cantão de Ticino, localizado no sul do país, 199 enfermeiros empreendiam em áreas como atenção domiciliar, tratamento de feridas, podologia, cuidados oncológicos e paliativos, obstetrícia, aleitamento e geriatria, oferecendo desde cuidados básicos até serviços especializados, com opções de atendimento 24 horas ou consultas programadas^(34)^.

Na Itália, as regulamentações garantem autonomia plena aos enfermeiros para organizar e executar suas atividades de forma autônoma, assim permitindo tanto práticas individuais quanto a formação de associações e cooperativas^(35)^. Ainda, o sistema oferece cobertura previdenciária específica para enfermeiros autônomos, fortalecendo o reconhecimento profissional e incentivando o empreendedorismo^(36)^. Além disso, uma plataforma desenvolvida pela Federação Nacional das Ordens das Profissões de Enfermagem conecta cidadãos a enfermeiros autônomos, facilitando o acesso aos serviços como atenção domiciliar, administração de medicamentos e tratamento de feridas, promovendo visibilidade e valorização da categoria^(37)^.

Já no Brasil, a Resolução COFEN nº. 568/2018, que autoriza o funcionamento de clínicas e consultórios de enfermagem, representa um marco regulatório importante para ampliar as oportunidades de empreendedorismo na profissão. Essa regulamentação encontra paralelos em outros países, como a Autoridade Plena de Prática (*Full Practice Authority* - FPA) implementada nos Estados Unidos, que concede aos enfermeiros especialistas autonomia para diagnosticar, iniciar e gerenciar tratamentos, incluindo a prescrição de medicamentos e substâncias controladas^(38)^. Nos estados norte-americanos que adotaram a FPA, houve um aumento de 374,1% na probabilidade de práticas autônomas, além de maior presença desses profissionais em áreas com escassez de cuidados primários em saúde^(39)^.

Nas Filipinas, o projeto *EntrepreNurse*, lançado em 2010, exemplifica o impacto de políticas públicas no fomento ao empreendedorismo. O programa incentivou enfermeiros a criarem negócios próprios, especialmente em regiões com escassez de profissionais, fornecendo financiamento, treinamento empresarial e suporte contratual. Como resultado, enfermeiros abriram empresas de assistência domiciliar e clínicas de reabilitação^(40)^.

De forma semelhante, na Tailândia, o *National Health Security Office* (NHSO) implementou, em 2019, uma política de saúde que estabeleceu mais de 100 clínicas de enfermagem e obstetrícia para ampliar o acesso aos serviços primários em comunidades rurais, reduzindo a sobrecarga hospitalar e fortalecendo a autonomia dos enfermeiros como líderes e gestores de serviços de saúde^(41)^.

Embora os contextos regulatórios variem, todas essas iniciativas demonstram como mudanças normativas ampliam o escopo de atuação dos enfermeiros, fomentam o empreendedorismo e melhoram o acesso à saúde. Contudo, o desenvolvimento do empreendedorismo na enfermagem enfrenta desafios, como políticas de saúde, financiamento, sistemas educacionais, papéis de gênero, localização geográfica, além de barreiras ético-legais, como legislações específicas de cada estado ou país, que podem tanto limitar quanto ampliar as oportunidades de prática autônoma na enfermagem^(42-45)^.

Globalmente, o empreendedorismo na enfermagem é ainda pouco expressivo, abrangendo apenas 0,5% a 1% dos profissionais^(46)^. No entanto, o potencial de expansão é significativo, especialmente com a inclusão de conteúdos sobre empreendedorismo nos currículos de enfermagem, capacitando os profissionais para superarem barreiras estruturais e culturais e a desenvolverem modelos de cuidado inovadores e sustentáveis^(47)^. Usando suas habilidades e experiências, enfermeiros empreendedores podem assumir papéis de liderança, mas essa área ainda demanda maior embasamento científico e presença nos conteúdos curriculares da formação em enfermagem^(41)^.

Nesse viés, o empreendedorismo pode ser também um caminho para resolução de problemas do passado e possibilidades de um futuro sustentável aos serviços e às pessoas, em particular a nova geração de profissionais de enfermagem, tendo relação com o Objetivo de Desenvolvimento Sustentável 8, de promover o crescimento econômico sustentado, inclusivo e sustentável, emprego pleno e produtivo, bem como trabalho decente para todas e todos^(48)^. Neste sentido, a sustentabilidade por meio do empreendedorismo não tem somente potencial para atender as demandas básicas de saúde da população, mas de produzir riqueza e empregos.

Ainda, a definição da Organização Pan-Americana da Saúde (OPAS) destaca a atenção primária à saúde (APS) como o primeiro ponto de contato capaz de atender até 90% das necessidades de saúde de uma pessoa, abrangendo desde promoção e prevenção (como vacinação) até tratamento, cuidados paliativos e reabilitação^(49)^. Ao comparar essa definição com os tipos de estabelecimentos e atividades principais descritos no CNES, é possível que se estabeleça uma relação clara entre a APS e o empreendedorismo de negócios na enfermagem.

À guisa de conclusão, ressalta-se que este estudo apresenta algumas limitações que precisam ser consideradas. Primeiro, a análise foi baseada em uma única base de dados, o que pode limitar a abrangência dos resultados. Além disso, a escassez de estudos semelhantes na área de enfermagem dificulta a comparação e a contextualização dos achados, reforçando a necessidade de ampliar as investigações sobre o tema em níveis nacional e internacional.

Outra limitação refere-se à impossibilidade de confirmar, nos casos de estabelecimentos registrados como Empresário Individual, se o enfermeiro listado como gerente ou administrador no CNES é também o proprietário do negócio. Essa estrutura jurídica não possui quadro societário, vinculando o CNPJ diretamente ao titular da empresa. Isso inviabiliza a verificação por meio da REDESIM e dificulta a distinção entre enfermeiros que são proprietários e aqueles contratados para gerenciar o estabelecimento.

Por conseguinte, essa questão destaca a importância de futuras investigações que adotem métodos de análise em nível individual, permitindo identificar com maior precisão a propriedade dos negócios e compreender de forma mais robusta a atuação empreendedora dos enfermeiros no Brasil. Outrossim, é necessário investigar se os tipos de estabelecimentos, as atividades principais e o nível de atenção realmente se concentram na média complexidade, como sugerem os dados analisados.

Apesar dessas limitações, este estudo se destaca como o primeiro, consoante o disponível na literatura, a caracterizar e descrever a distribuição espacial e temporal dos estabelecimentos assistenciais de enfermagem em nível nacional no Brasil. Consequentemente, os resultados oferecem dados inéditos sobre o empreendedorismo de negócios em enfermagem, ampliando a compreensão do tema e fornecendo uma base sólida para futuras investigações.

## Conclusão

Por fim, observa-se um crescimento expressivo nos cadastros, especialmente após a publicação de resoluções do COFEN que incentivam a prática autônoma e liberal na enfermagem, ampliando o escopo de atuação dos enfermeiros e promovendo oportunidades para o empreendedorismo na área. A formalização e o registro no CNES representam um avanço na profissionalização da gestão em enfermagem, sendo essenciais para garantir benefícios como convênios com planos de saúde, reconhecimento profissional e incentivos fiscais. Somente com registros formais e um panorama consolidado é possível fortalecer a profissão e criar um ambiente favorável ao desenvolvimento sustentável do setor.

# Translation: Emprendimiento de negocios en establecimientos asistenciales de enfermería en Brasil: un estudio ecológico

**DOI:** 10.1590/1518-8345.7657.4583

**Authors:** Matheus Moraes Silva, Jouhanna do Carmo Menegaz, Glenda Roberta Oliveira Naiff Ferreira, Josele de Jesus Quaresma Trindade Costa, Maria Eduarda Libório Martins, Allison Squires

**Affiliations:** 1 Universidade Federal do Pará, Faculdade de Enfermagem, Belém, PA, Brasil.; 2 Becaria (o) de la Coordenação de Aperfeiçoamento de Pessoal de Nível Superior (CAPES), Brasil.; 3 Universidade do Estado de Santa Catarina, Departamento de Enfermagem, Chapecó, SC, Brasil.; 4 Becaria del Conselho Nacional de Desenvolvimento Científico e Tecnológico (CNPq), Brasil.; 5 New York University, Rory Meyers College of Nursing, New York, NY, Estados Unidos de América.

**Keywords:** Emprendimiento, Servicios de Enfermería, Mercado de Trabajo, Práctica Privada de Enfermería, Autonomía Profesional, Cobertura Universal de Salud

## Abstract

caracterizar y describir la distribución espacial y temporal de los establecimientos asistenciales de enfermería en Brasil según el *Cadastro Nacional de Estabelecimentos de Saúde*.

estudio ecológico, utilizando datos secundarios del *Cadastro Nacional de Estabelecimentos de Saúde*, agregados por año, estado y región. Las variables extraídas fueron: año de registro, sexo del enfermero gerente o administrador, número de enfermeros por establecimiento, tipo de establecimiento, actividad principal, nivel de atención, naturaleza jurídica y convenio. Se realizó estadística descriptiva y análisis temporal en el programa *Joinpoint*.

entre 2003 y 2023 se encontraron 340 establecimientos de enfermería con registro activo. Las regiones Sur y Sudeste concentraron la mayor parte de los establecimientos. Hubo predominio de consultorios, clínicas especializadas y servicios de atención domiciliaria. El análisis temporal indicó que entre 2003 y 2021, hubo un crecimiento anual del 30,07 % (*p*= 0,00). Considerando el período total (2003-2023), hubo un crecimiento anual del 25,01 %, indicando una tendencia creciente de los emprendimientos a lo largo de los años.

la formalización y registro reflejan la profesionalización de la gestión en enfermería. Se observa un crecimiento en los registros tras la publicación de resoluciones del *Conselho Federal de Enfermagem* que apoyan la práctica autónoma y/o liberal.

## Destacados

**(1)** Las regiones Sur y Sudeste concentran más establecimientos de enfermería. **(2)** Entre 2003 y 2023 hubo un crecimiento anual del 25,01 % en los emprendimientos. **(3)** Predominan los consultorios aislados y las clínicas especializadas. **(4)** Las actividades principales son: consulta ambulatoria, atención domiciliaria e inmunización. **(5)** La mayoría acepta pago particular y planes de salud privados.

## Introducción

El emprendimiento en enfermería se define como la habilidad de identificar oportunidades, desarrollar proyectos innovadores e implementar soluciones para la mejora continua del cuidado en salud. El concepto va más allá de la creación de empresas, abarcando innovación, investigación y avance científico, permitiendo que los enfermeros transformen prácticas de salud y respondan a las demandas de la sociedad^(1)^.

En el escenario internacional, el emprendimiento en enfermería ha sido ampliamente reconocido por su potencial para enfrentar desafíos complejos y promover innovaciones en la prestación de servicios de salud^(2)^. Una revisión sistemática destacó que enfermeros emprendedores identifican brechas en los sistemas de salud y desarrollan servicios innovadores, como clínicas especializadas, empresas de atención domiciliaria y consultorios independientes, diversificando los servicios y ampliando el acceso a los cuidados^(3)^.

En 2023, la *American Nurses Association Enterprise Innovation* destacó que más de 300 enfermeros emprendedores lideraron iniciativas como agentes de cambio. Estas acciones incluyeron el desarrollo de dispositivos médicos, nuevos modelos en la prestación de cuidados, creación de negocios, investigación y especialidades educacionales, evidenciando el papel del emprendimiento en el avance de la profesión^(4)^.

En Brasil, el sector de la salud está preparado para una expansión impulsada por cambios demográficos, políticos y transformaciones en las actitudes de los consumidores. El envejecimiento de la población, el aumento de las enfermedades crónicas y el crecimiento de la clase media han generado una demanda creciente por servicios y tecnologías de salud avanzadas^(5-7)^. Además, el creciente interés de los brasileños por la salud preventiva está impulsando el desarrollo de subsectores, como clínicas de salud y bienestar^(8)^.

Este contexto destaca oportunidades emprendedoras en el campo de la enfermería. El derecho de emprender y tener un negocio propio en enfermería está respaldado por la Ley del Ejercicio Profesional nº 7.498/1986, que asegura la autonomía del enfermero. Además, el emprendimiento de negocios en enfermería se destaca en el escenario brasileño, sobre todo, con la Resolución nº 568/2018 del *Conselho Federal de Enfermagem* (COFEN), que reglamenta la actuación de enfermeros en clínicas y consultorios de enfermería^(9)^.

Así, para los propósitos de esta resolución, la clínica de enfermería se define como un establecimiento constituido por consultorios y ambientes destinados a la atención de enfermería individual, colectiva y/o domiciliaria. Por otro lado, el consultorio de enfermería se define como el área física exclusiva para consultas y otras actividades privativas del enfermero^(9)^. Tales definiciones refuerzan el derecho de emprender y ejercer la profesión de enfermería de manera autónoma e independiente.

El emprendedor de negocios en enfermería que posee un establecimiento asistencial, entendido en este estudio como clínicas o consultorios, necesita cumplir con una serie de requisitos jurídicos, tributarios, sanitarios y regulatorios ante el *Conselho Regional de Enfermagem* para actuar de forma ética y legal^(10)^. Los establecimientos asistenciales son espacios físicos delimitados y permanentes en los cuales se realizan acciones y servicios de salud humana bajo responsabilidad técnica^(11)^.

En el contexto brasileño, la gestión de los establecimientos asistenciales está normatizada por el *Cadastro Nacional de Estabelecimentos de Saúde* (CNES), sistema informatizado mantenido por el Ministerio de Salud que centraliza información de todos los servicios de salud en el país, públicos o privados, con o sin convenio con el *Sistema Único de Saúde* (SUS), siendo obligatorio que todos los establecimientos que ofrezcan atención a la salud humana posean un registro^(11)^.

Considerando el poco conocimiento sobre el emprendimiento de negocios en establecimientos asistenciales de enfermería, la escasez de estudios que mapeen sus características y tendencias temporales en Brasil, y la ausencia de datos unificados disponibles por los *Conselhos Regionais de Enfermagem* (CORENs) y por el COFEN sobre el panorama de estos emprendimientos, este estudio tuvo como objetivo caracterizar y describir la distribución espacial y temporal de los establecimientos asistenciales de enfermería en Brasil según el CNES.

## Método

### Diseño del estudio

Se trata de un estudio ecológico, guiado por la herramienta *Strengthening the Reporting of Observational Studies in Epidemiology* (STROBE). Los análisis y resultados de estudios ecológicos son aplicables únicamente a nivel poblacional^(12)^. En este estudio, las unidades de análisis fueron los 27 estados y las 5 (cinco) regiones administrativas de Brasil.

### Escenario

El estudio se llevó a cabo en el territorio brasileño, que posee una extensión territorial de 8.510.417,77 km², dividida en 26 estados, un Distrito Federal y 5.570 municipios. El país está organizado en cinco macrorregiones: Norte, Nordeste, Centro-Oeste, Sudeste y Sur. Hasta agosto de 2022, la población brasileña se estimaba en 203.080.756 individuos^(13)^.

Según datos del COFEN, el país cuenta con aproximadamente 756.054 profesionales enfermeros registrados hasta noviembre de 2024^(14)^. En cuanto a la proporción de enfermeros por 1.000 habitantes en las macrorregiones de Brasil, un estudio realizado en 2021, basado en datos del perfil de la enfermería publicado por el COFEN en 2016, presentó las siguientes estadísticas: Región Norte (1,6 por 1.000 habitantes), Región Nordeste (1,76 por 1.000 habitantes), Región Centro-Oeste (2,25 por 1.000 habitantes), Región Sudeste (2,38 por 1.000 habitantes) y Región Sur (1,77 por 1.000 habitantes)^(15)^.

### Población y criterios de selección

Se incluyeron los datos de los establecimientos asistenciales de enfermería clasificados como Entidades Empresariales y Personas Físicas (grupo), los que el profesional enfermero actuaba como gerente o administrador, considerando solo aquellos con registro activo en el CNES. Se excluyeron los datos que presentaban información incompleta o que no cumplían con los requisitos preestablecidos.

Cabe destacar que no se definió ningún recorte temporal para la selección de los datos, con el objetivo de integrar el mayor número de establecimientos y dimensionar adecuadamente la evolución longitudinal del emprendimiento en establecimientos asistenciales de enfermería a lo largo del tiempo.

### Recolección de datos

Para la recolección de datos se utilizó la plataforma del CNES, en la sección de Consulta de Establecimiento, empleando las palabras clave: “Clínica de Enfermagem”, “Consultório de Enfermagem”, “Enfermeiro”, “Enfermeira”, “*Nursing*” y “*Nurse*”. Esta metodología de búsqueda se justifica por la ausencia de una opción que permita buscar los establecimientos por categoría profesional de salud. De este modo, se optó por esta estrategia con el fin de filtrar los establecimientos relacionados con la enfermería por el nombre comercial o empresarial.

Como el CNES no especifica si el profesional listado como gerente o administrador también es propietario del establecimiento, se realizó una verificación cruzada utilizando la plataforma de la *Rede Nacional para a Simplificação do Registro e da Legalização de Empresas e Negócios* (REDESIM), en la sección de consulta del *Cadastro Nacional de Pessoa Jurídica* (CNPJ). Cada número de CNPJ de los establecimientos identificado en el CNES fue consultado, con el objetivo de verificar si el nombre del enfermero gerente o administrador constaba en el cuadro de socios de la empresa.

### Variables del estudio

La variable de este estudio ecológico fue una medida agregada, en la cual los datos de los establecimientos fueron agrupados por año, estado y región. Las variables extraídas fueron: año de registro, sexo del enfermero gerente o administrador, número de enfermeros por establecimiento, tipo de establecimiento, actividad principal, nivel de atención, naturaleza jurídica y convenio.

En el análisis de tendencia, la variable dependiente fue el porcentaje de establecimientos (número de establecimientos por año, dividido por el total de establecimientos en el período, multiplicado por 100). La variable independiente fueron los años.

### Período del estudio

El estudio se llevó a cabo entre los meses de diciembre de 2023 y junio de 2024. El período de la serie histórica considerado en la recolección de datos fue de 2003 a 2023.

### Tratamiento y análisis de los datos

Los datos fueron almacenados en una hoja de cálculo de *Microsoft Excel*®, excluyendo inconsistencias tras la doble verificación (duplicidad de registro). La descripción se presentó utilizando frecuencias absolutas (n) y relativas (%). El número de enfermeros registrados en los establecimientos se presentó por media y desviación estándar. Los datos fueron presentados en tablas y mapas. Los mapas coropléticos fueron generados usando la versión 3.34.3 del *software* QGIS, presentando la frecuencia absoluta y relativa de los establecimientos por estado, utilizando el mismo cálculo realizado en la variable dependiente del análisis de tendencia.

El estudio se caracteriza como una serie temporal al utilizar una secuencia de datos en intervalos regulares, correspondiente al porcentaje de establecimientos por año, en el período de 2003 a 2023. Una serie histórica está compuesta por tres componentes, siendo el análisis de tendencia uno de ellos^(16)^. El modelo de regresión *Joinpoint* fue adoptado por su capacidad de identificar uno o más cambios en la tendencia lineal en escala logarítmica^(17)^.

El análisis de tendencia se realizó utilizando el *software Joinpoint Trend Analysis*, versión 5.3.0, desarrollado por el *National Cancer Institute* (Calverton, Estados Unidos de América). Se adoptaron los siguientes parámetros: los valores de la serie (porcentaje de establecimientos en cada año) fueron definidos como variable dependiente (Y), mientras que los años del período analizado (2003 a 2023) fueron considerados como variable independiente (X). El Intervalo de Confianza (IC) fue del 95% y el valor de *p* < 0,05.

La opción “Errores heterocedásticos/correlacionados” fue configurada como “Error Estándar”, y se ajustó un modelo de errores no correlacionados. Esta es la selección estándar, que considera que los errores aleatorios poseen varianza no constante (son heterocedásticos) y estima los coeficientes de regresión mediante el método de mínimos cuadrados ponderados (*Weighted Least Squares* – WLS)^(18)^. La elección del modelo de regresión *Joinpoint* más adecuado se hizo mediante la prueba de permutación Monte Carlo, con 4.499 permutaciones. Este método permite identificar cuánto necesita aumentar una proporción para ser estadísticamente significativa^(17)^.

Para describir las tendencias identificadas, se utilizaron dos indicadores: la Variación Porcentual Anual (*Annual Percent Change* – APC), que presupone que la proporción de establecimientos cambia a un porcentaje constante en relación con el año anterior y varía linealmente en escala logarítmica^(19)^; y la Variación Porcentual Anual Promedio (*Average Annual Percentage Change* – AAPC), que es una medida que resume la tendencia en un intervalo fijo preestablecido. Esta métrica permite describir los APC medios a lo largo de varios años utilizando un solo valor. El cálculo de la AAPC se realiza como un promedio ponderado de los APC del modelo, con pesos correspondientes a la longitud del intervalo de cada APC^(20)^.

Las tendencias fueron clasificadas como crecientes o decrecientes cuando los valores de APC y AAPC eran positivos o negativos, respectivamente, y presentaban significancia estadística establecida en *p* < 0,05. En ausencia de significancia (*p* > 0,05), la tendencia fue clasificada como estacionaria^(21)^.

### Aspectos éticos

Este estudio cuenta con la aprobación del Comité de Ética e Investigación de la *Universidade do Estado de Santa Catarina*, bajo el dictamen número 5.440.353.

## Resultados

Entre los años 2003 y 2023, se encontraron 340 establecimientos de enfermería con registro activo en el CNES, distribuidos de la siguiente manera en las macrorregiones brasileñas: 17 (5,0%) en el Norte, 50 (14,71%) en el Nordeste, 31 (9,12%) en el Centro-Oeste, 137 (40,29%) en el Sudeste y 105 (30,88%) en el Sur. En cuanto al sexo del enfermero gerente o administrador, 284 (83,53 %) eran del sexo femenino y 56 (16,47%) del sexo masculino. En relación con el número de enfermeros registrados por establecimiento, la media fue de 1,57, con una desviación estándar de ± 2,67, con un mínimo de 1 y un máximo de 45 enfermeros.

En la Tabla 1 se presentan las características de los establecimientos de enfermería. Con respecto al tipo de establecimiento, los consultorios aislados son los más comunes, representando 128 (37,64%) de los emprendimientos, seguidos por las clínicas especializadas (n=82; 24,12%), los servicios de atención domiciliaria (n=74; 21,76%) y los centros de inmunización (n=31; 9,12%). En cuanto a las principales actividades desarrolladas por los enfermeros, se destaca la consulta ambulatoria (n=197; 57,94%), seguida por la atención domiciliaria e inmunización, con 63 (18,53%) y 46 (13,53%) respectivamente, como se muestra en la Tabla 1.

**Tabla 1 -:** Características de los establecimientos de enfermería, en el período de 2003-2023 (n = 340). Brasil, 2024

**Variable**		**Regiones de Brasil**					
		Norte 17 (%)	Nordeste 50 (%)	Centro-oeste 31 (%)	Sudeste 137 (%)	Sur 105 (%)	Brasil 340 (%)*
**Tipo de establecimiento**	Centro de inmunización	0 (0,00)	4 (8,00)	4 (12,90)	20 (14,60)	3 (2,86)	31 (9,12)
	Clínica especializada	4 (23,53)	26 (52,00	12 (38,72)	24 (17,52)	16 (15,24)	82 (24,12)
	Consultorio aislado	7 (41,18)	9 (18,00)	9 (29,03)	42 (30,65)	61 (58,10)	128 (37,64)
	Cooperativa	1 (5,88)	2 (4,00)	2 (6,45)	1 (0,73)	5 (4,76)	11 (3,24)
	Policlínica	1 (5,88)	0 (0,00)	0 (0,00)	2 (1,46)	2 (1,90)	5 (1,47)
	Servicio de atención domiciliaria ( *Home care* )	4 (23,53)	9 (18,00)	4 (12,90)	43 (31,39)	14 (13,33)	74 (21,76)
	Unidad de apoyo diagnóstico y terapia	0 (0,00)	0 (0,00)	0 (0,00)	5 (3,65)	4 (3,81)	9 (2,65)
**Actividad principal**	Administración	1 (5,88)	2 (4,00)	2 (6,45)	1 (0,73)	5 (4,76)	11 (3,24)
	Asistencia en salud	3 (17,65)	5 (10,00)	1 (3,23)	4 (2,92)	7 (6,67)	20 (5,88)
	Atención domiciliaria	3 (17,65)	6 (12,00)	3 (9,68)	38 (27,74)	13 (12,38)	63 (18,53)
	Consulta ambulatoria	9 (52,94)	29 (58,00)	20 (64,51)	66 (48,17)	73 (69,52)	197 (57,94)
	Hospitalidad	0 (0,00)	0 (0,00)	0 (0,00)	1 (0,73)	2 (1,91)	3 (0,88)
	Inmunización	1 (5,88)	8 (16,00)	5 (16,13)	27 (19,71)	5 (4,76)	46 (13,53)
**Nivel de atención**	Atención básica	4 (23,53)	4 (8,00)	3 (9,68)	8 (5,84)	41 (39,05)	60 (17,65)
	Media complejidad	13 (76,47)	46 (92,00)	28 (90,32)	128 (93,43)	63 (60,00)	278 (81,76)
	Alta complejidad	0 (0,00)	0 (0,00)	0 (0,0)	1 (0,73)	1 (0,95)	2 (0,59)
**Naturaleza jurídica**	Cooperativa	1 (5,88)	2 (4,00)	2 (6,45)	1 (0,73)	5 (4,76)	11 (3,24)
	Empresario Individual	5 (29,41)	9 (18,00)	5 (16,13)	17 (12,41)	24 (22,86)	60 (17,65)
	Sociedad empresaria limitada	11 (64,71)	37 (74,00)	24 (77,42)	111 (81,02)	72 (68,57)	255 (75,00)
	Sociedad simple limitada	0 (0,00)	1 (2,00)	0 (0,00)	6 (4,38)	4 (3,81)	11 (3,24)
	Sociedad simple pura	0 (0,00)	1 (2,00)	0 (0,00)	2 (1,46)	0 (0,00)	3 (0,87)
**Convenio**	Particular	15 (88,24)	25 (50,00)	18 (58,06)	69 (50,36)	69 (65,71)	196 (57,65)
	Plan de salud privado y particular	2 (11,76)	23 (46,00)	13 (41,94)	64 (46,72)	32 (30,48)	134 (39,41)
	Particular, SUS ^†^ y plan de salud privado.	0 (0,0)	2 (4,00)	0 (0,00)	4 (2,92)	4 (3,81)	10 (2,94)

Fuente: *Cadastro Nacional de Estabelecimentos de Saúde* (CNES) *% = Frecuencia Relativa; ^†^ SUS = *Sistema Único de Saúde*

Al analizar los niveles de atención en salud prestados, se observa una distribución diversificada en términos de la complejidad de los servicios ofrecidos. Así, la mayoría de las atenciones está relacionada con la complejidad media (n=278; 81,76%), seguida por la atención básica (n=60; 17,65%). La presencia de atenciones de alta complejidad es mínima, representando apenas el 0,59% de los emprendimientos (Tabla 1). En relación con la naturaleza jurídica, la sociedad empresaria limitada es predominante en la mayoría de los tipos de establecimientos (n=255; 75,00%), seguida por el empresario individual (n=60; 17,65%). Otras categorías jurídicas, como sociedades simples limitadas o sociedades simples puras, tienen una presencia menos significativa (Tabla 1).

Además, al analizar los tipos de convenios disponibles para el pago de los servicios prestados, se observa que la mayoría acepta exclusivamente pago particular (n=196; 57,65%). Por otro lado, una proporción considerable (n=134; 39,41%) de los establecimientos permite el uso tanto de planes de salud privados como de pago particular, mientras que una menor proporción (n=10; 2,94%) atiende mediante pago particular, plan de salud privado y por el SUS (Tabla 1).

En la Figura 1 se presentan, respectivamente, mapas con la distribución de los establecimientos en las unidades federativas, en términos de frecuencia absoluta (Figura 1 A) y relativa (Figura 1 B). En este contexto, se destacan Rio Grande do Sul, Santa Catarina, São Paulo, Minas Gerais y Rio de Janeiro por la cobertura más amplia de servicios autónomos de enfermería en comparación con otras áreas del país. Estos estados poseen un número de establecimientos que varía de 20 a 70, representando un rango porcentual del 5,79% al 20,59%.

Además, también merecen destaque Mato Grosso, Bahía, Paraíba y Paraná, con un número de entre 13 y 20 establecimientos, lo que corresponde a un rango porcentual entre el 3,92% y el 5,79% (Figura 1 A y Figura 1 B). La región Norte presentó el menor porcentaje de establecimientos de enfermería, con apenas 17, representando el 5,0% del valor total.

**Figura 1 -:** Distribución espacial de los establecimientos de enfermería en las unidades federativas entre 2003-2023 (n = 340). Brasil, 2024

*A= Frecuencia absoluta de establecimientos de enfermería; ^†^ B = Frecuencia relativa de establecimientos de enfermería

La Tabla 2 presenta la distribución temporal de los establecimientos de enfermería en el período de 2003 a 2023. Se observa que en el año 2021 hubo el mayor número de establecimientos registrados.

**Tabla 2 -:** Distribución temporal de establecimientos de enfermería, 2003-2023. Brasil, 2024

**A** ñ **o**	**N** *	**%** ^†^	**Error-estándar** ^‡^
2003	1	0,29	0,29
2004	1	0,29	0,29
2005	1	0,29	0,29
2006	1	0,29	0,29
2007	2	0,59	0,41
2008	3	0,88	0,50
2009	2	0,59	0,41
2010	2	0,59	0,41
2011	6	1,76	0,71
2012	6	1,76	0,71
2013	6	1,76	0,71
2014	9	2,65	0,87
2015	12	3,53	1,00
2016	13	3,82	1,04
2017	16	4,71	1,14
2018	28	8,24	1,49
2019	31	9,12	1,56
2020	46	13,53	1,85
2021	63	18,53	2,10
2022	47	13,82	1,87
2023	46	13,53	1,85

*N= Frecuencia absoluta; ^†^% = Frecuencia relativa; ^‡^ Error-estándar en cada período (año)

La Tabla 3 presenta el análisis de tendencia temporal del emprendimiento en establecimientos de enfermería en Brasil. Durante los 20 años de estudio, se observaron dos períodos distintos. Entre 2003 y 2021, el análisis reveló una tendencia creciente, expresada por la APC del 30,07% anual (IC 95%: 28,51-34,65), con un valor de p = 0,00. Sin embargo, de 2021 a 2023, el análisis indicó una variación porcentual negativa, aunque no significativa, de -12,45% (IC 95%: -24,09; 1,67; *p* = 0,17) (Tabla 3).

**Tabla 3 -:** Análisis de tendencia temporal de los establecimientos de enfermería, 2003-2023. Brasil, 2024

**Período**	APC*	p **- valor** ^†^	**IC 95%** ^‡^	**Tendencia**
2003 – 2021	30,07	0,00	28,51 – 34,65	Creciente
2021 – 2023	-12,45	0,17	-24,09 – 1,67	Estacionaria
2003 – 2023	25,01 ^§^	0,00	23,55 – 28,65	Creciente

*APC = Variación Porcentual Anual; ^†^ *p* < 0,05; ^‡^ IC 95% = Intervalo de Confianza de 95%; ^§^ AAPC = Variación Porcentual Anual Promedio

Al analizar el período total (2003-2023), la AAPC fue del 25,01% (IC 95%: 23,55 – 28,65), con un *p*-valor = 0,00, indicando una tendencia creciente de estos emprendimientos a lo largo de esos años, como se muestra en la Tabla 3.

## Discusión

Los resultados de este estudio revelan aspectos importantes sobre la distribución espacial y temporal de los establecimientos asistenciales de enfermería en Brasil, así como la caracterización de los tipos de establecimientos, actividades principales y niveles de atención en salud.

En cuanto a la distribución de los establecimientos en las regiones de Brasil, se observa una concentración en el Sudeste y el Sur. En un estudio sobre la distribución de fisioterapeutas realizado en el CNES, la región Sudeste también se destacó, con el 50% de los profesionales^(22)^. Aunque los datos de este estudio no puedan considerarse definitivos para ofrecer un panorama completo sobre el emprendimiento de negocios en establecimientos asistenciales de enfermería, no es inesperada la expresividad de la región Sudeste, considerando su desarrollo económico a lo largo de la historia de Brasil y la concentración de vacantes en cursos de grado^(23)^, así como la cantidad de profesionales de enfermería en el estado de São Paulo, que supera los 700 mil, de los cuales 183 mil son enfermeros^(24)^.

Se destaca la concentración de la actividad productiva brasileña en la región Sudeste, así como la centralización del poder político y el dominio económico, que llevaron a una rápida transición del modelo agroexportador al industrial, obteniendo ventajas duraderas en el proceso^(25)^. Desde la perspectiva del emprendimiento de negocios, se resalta la concentración del Producto Interno Bruto (PIB) en las regiones mencionadas, así como los mayores ingresos per cápita, que las convierten en entornos potencialmente favorables para el emprendimiento, al contar con un mercado consumidor activo, con los estados de São Paulo, Río de Janeiro, Rio Grande do Sul y Santa Catarina ocupando cuatro de las cinco primeras posiciones^(26)^.

Con respecto a la cantidad de registros a lo largo de los años, los datos muestran un crecimiento más significativo a partir de 2018, posiblemente influenciado por cambios en el contexto normativo de la profesión, como la Resolución COFEN nº 568/2018. Otras resoluciones, como la COFEN nº 685/2022, que instituye la Anotación de Responsabilidad Técnica (ART) en los servicios de enfermería autónomos y/o liberales, también pueden estar relacionadas con este aumento. En este contexto, también es pertinente mencionar que, en un período similar al de la recolección de datos, de 2005 a 2021, un estudio identificó que el COFEN publicó 385 resoluciones que respaldan la práctica de enfermería^(27)^.

Sin embargo, no hay evidencias que permitan establecer una relación causal directa entre estas normativas y el aumento observado en los datos. Otros factores, como el PIB y el Índice de Desarrollo Humano (IDH), también pueden haber influido en este crecimiento durante el período analizado. Por lo tanto, se requieren investigaciones futuras para evaluar no solo la eficacia de las resoluciones del COFEN, sino también el papel de estos factores en el fomento del emprendimiento de negocios en enfermería, permitiendo una comprensión más amplia de los determinantes del crecimiento de estos establecimientos en Brasil.

Aunque los datos sugieren crecimiento, es posible que no reflejen ni siquiera una pequeña parte de los establecimientos de enfermería, dado que no existe un panorama consolidado en los CORENs sobre esta realidad. Además, el registro en el CNES no es obligatorio para la inscripción de establecimientos en los consejos regionales de enfermería. Sin embargo, es un requisito para convenios con planes privados, que representan un significativo 39,41% de los datos. Aunque el enfermero, como profesional liberal, puede actuar únicamente con el Registro de Persona Física (CPF), existe un movimiento hacia la profesionalización de la gestión y la búsqueda de nuevos espacios para los cuales la formalización, como en el CNES, se hace necesaria.

Los tipos de establecimientos de enfermería predominantes en Brasil, según los datos del CNES, son consultorios aislados, clínicas especializadas y servicios de atención domiciliaria (*Home care*). Las principales actividades incluyen consultas ambulatorias, atención domiciliaria e inmunización, demostrando un enfoque en acciones de cuidado directo y preventivo. Sin embargo, la mayoría de estos establecimientos actúan en la atención secundaria en salud, orientados a servicios de complejidad media. En cuanto a la forma de pago, la mayoría es directa, de acuerdo con los servicios prestados o a través de planes de salud.

En el escenario internacional, se observa un mayor énfasis en los cuidados primarios^(28)^, especialmente en actividades como la práctica clínica privada y el *Home care* ^(29-31)^. En Canadá, los enfermeros autónomos se destacan en servicios especializados, como cuidados de heridas y consultoría en bienestar^(32)^. En Suiza, la práctica autónoma en enfermería está reglamentada, permitiendo la actuación en diversas especialidades. En 2016, la Asociación Suiza de Enfermeros (ASE) registró alrededor de 1.800 profesionales en esta modalidad^(33)^. Hasta octubre de 2024, en el cantón de Ticino, ubicado en el sur del país, 199 enfermeros emprendían en áreas como atención domiciliaria, tratamiento de heridas, podología, cuidados oncológicos y paliativos, obstetricia, lactancia y geriatría, ofreciendo desde cuidados básicos hasta servicios especializados, con opciones de atención las 24 horas o consultas programadas^(34)^.

En Italia, las regulaciones garantizan plena autonomía a los enfermeros para organizar y ejecutar sus actividades de forma independiente, permitiendo tanto prácticas individuales como la formación de asociaciones y cooperativas^(35)^. El sistema también ofrece cobertura previsional específica para enfermeros autónomos, fortaleciendo el reconocimiento profesional e incentivando el emprendimiento^(36)^. Además, una plataforma desarrollada por la Federación Nacional de las Órdenes de las Profesiones de Enfermería conecta a los ciudadanos con enfermeros autónomos, facilitando el acceso a servicios como atención domiciliaria, administración de medicamentos y tratamiento de heridas, promoviendo la visibilidad y valorización de la categoría^(37)^.

En Brasil, la Resolución COFEN nº 568/2018, que autoriza el funcionamiento de clínicas y consultorios de enfermería, representa un hito regulatorio importante para ampliar las oportunidades de emprendimiento en la profesión. Esta regulación encuentra paralelismos en otros países, como la *Full Practice Authority* (FPA) implementada en Estados Unidos, que otorga a los enfermeros especialistas autonomía para diagnosticar, iniciar y gestionar tratamientos, incluyendo la prescripción de medicamentos y sustancias controladas^(38)^. En los estados estadounidenses que adoptaron la FPA, hubo un aumento del 374,1% en la probabilidad de prácticas autónomas, además de una mayor presencia de estos profesionales en áreas con escasez de cuidados primarios de salud^(39)^.

En Filipinas, el proyecto *EntrepreNurse*, lanzado en 2010, ejemplifica el impacto de las políticas públicas en el fomento del emprendimiento. El programa incentivó a los enfermeros a crear sus propios negocios, especialmente en regiones con escasez de profesionales, proporcionando financiamiento, capacitación empresarial y apoyo contractual. Como resultado, los enfermeros abrieron empresas de asistencia domiciliaria y clínicas de rehabilitación^(40)^.

De manera similar, en Tailandia, la *National Health Security Office* (NHSO) implementó, en 2019, una política de salud que estableció más de 100 clínicas de enfermería y obstetricia para ampliar el acceso a servicios primarios en comunidades rurales, reduciendo la sobrecarga hospitalaria y fortaleciendo la autonomía de los enfermeros como líderes y gestores de servicios de salud^(41)^.

Aunque los contextos regulatorios varían, todas estas iniciativas demuestran cómo los cambios normativos amplían el alcance de actuación de los enfermeros, fomentan el emprendimiento y mejoran el acceso a la salud. Sin embargo, el desarrollo del emprendimiento en la enfermería enfrenta desafíos, como políticas de salud, financiamiento, sistemas educativos, roles de género, ubicación geográfica, además de barreras ético-legales, como legislaciones específicas de cada estado o país, que pueden tanto limitar como ampliar las oportunidades de práctica autónoma en la enfermería^(42-45)^.

A nivel global, el emprendimiento en enfermería sigue siendo poco significativo, abarcando apenas entre el 0,5% y el 1% de los profesionales^(46)^. No obstante, el potencial de expansión es considerable, especialmente con la inclusión de contenidos sobre emprendimiento en los planes de estudio de enfermería, capacitando a los profesionales para superar barreras estructurales y culturales y desarrollar modelos de cuidado innovadores y sostenibles^(47)^. Usando sus habilidades y experiencias, los enfermeros emprendedores pueden asumir roles de liderazgo, pero esta área aún demanda mayor fundamentación científica y presencia en los contenidos curriculares de la formación en enfermería^(41)^.

El emprendimiento también puede ser un camino para resolver problemas del pasado y ofrecer posibilidades de un futuro sostenible para los servicios y las personas, en particular para la nueva generación de profesionales de enfermería, teniendo relación con el Objetivo de Desarrollo Sostenible 8, que busca promover el crecimiento económico sostenido, inclusivo y sostenible, el empleo pleno y productivo y el trabajo decente para todos y todas^(48)^. En este sentido, la sostenibilidad a través del emprendimiento no solo tiene potencial para atender las demandas básicas de salud de la población, sino también para generar riqueza y empleo.

La definición de la Organización Panamericana de la Salud (OPS) destaca la atención primaria de salud (APS) como el primer punto de contacto capaz de atender hasta el 90% de las necesidades de salud de una persona, abarcando desde la promoción y prevención (como la vacunación) hasta el tratamiento, cuidados paliativos y rehabilitación^(49)^. Al comparar esta definición con los tipos de establecimientos y actividades principales descritos en el CNES, es posible establecer una relación clara entre la APS y el emprendimiento de negocios en la enfermería.

Este estudio presenta algunas limitaciones que deben considerarse. En primer lugar, el análisis se basó en una única base de datos, lo que puede limitar el alcance de los resultados. Además, la escasez de estudios similares en el área de enfermería dificulta la comparación y contextualización de los hallazgos, reforzando la necesidad de ampliar las investigaciones sobre el tema a nivel nacional e internacional.

Otra limitación se refiere a la imposibilidad de confirmar, en los casos de establecimientos registrados como Empresario Individual, si el enfermero listado como gerente o administrador en el CNES es también el propietario del negocio. Esta estructura jurídica no posee un cuadro societario, vinculando el CNPJ directamente al titular de la empresa. Esto imposibilita la verificación a través de REDESIM y dificulta la distinción entre enfermeros que son propietarios y aquellos contratados para gestionar el establecimiento.

Esta cuestión destaca la importancia de futuras investigaciones que adopten métodos de análisis a nivel individual, permitiendo identificar con mayor precisión la propiedad de los negocios y comprender de forma más robusta la actuación emprendedora de los enfermeros en Brasil. Además, es necesario investigar si los tipos de establecimientos, las actividades principales y el nivel de atención realmente se concentran en la media complejidad, como sugieren los datos analizados.

A pesar de estas limitaciones, este estudio se destaca como el primero, según la literatura disponible, en caracterizar y describir la distribución espacial y temporal de los establecimientos asistenciales de enfermería a nivel nacional en Brasil. Los resultados ofrecen datos inéditos sobre el emprendimiento de negocios en enfermería, ampliando la comprensión del tema y proporcionando una base sólida para futuras investigaciones.

## Conclusión

Se observa un crecimiento significativo en los registros, especialmente tras la publicación de resoluciones del COFEN que incentivan la práctica autónoma y liberal en la enfermería, ampliando el alcance de actuación de los enfermeros y promoviendo oportunidades para el emprendimiento en el área. La formalización y el registro en el CNES representan un avance en la profesionalización de la gestión en enfermería, siendo esenciales para garantizar beneficios como convenios con planes de salud, reconocimiento profesional e incentivos fiscales. Solo con registros formales y un panorama consolidado es posible fortalecer la profesión y crear un entorno favorable para el desarrollo sostenible del sector.
